# Composition-engineered Sr_1−x_Ba_x_TiO_3_ for high-efficiency dielectric resonator antennas in 5G/6G bands

**DOI:** 10.1038/s41598-026-61044-1

**Published:** 2026-07-14

**Authors:** Moustafa A. Darwish, F. Fakhry, Marwa M. Hussein, Yousef M. Abd El-Maboud, Enas H. El-Ghazzawy, Anwer S. Abd El-Hameed, Asmaa I. Afifi, Sherif G. Elsharkawy, M. M. Salem

**Affiliations:** 1 Physics Department, Faculty of Science, Tanta University, Tanta, 31527 Egypt; 2 Electronics Research Institute, Giza, 12622 Egypt; 3 Basic and Applied Sciences, College of Engineering and Technology, AASTMT, Alexandria, 1029 Egypt

**Keywords:** Sr_1−x_Ba_x_TiO_3_ nanoparticles, Ferroelectric perovskites, Microwave dielectric properties, Dielectric resonator antennas (DRAs), Sub-6 GHz (5G/6G), Engineering, Materials science, Nanoscience and technology, Physics

## Abstract

We report Sr_1−x_Ba_x_TiO_3_ (x = 0.0–0.4) nanoparticles synthesized by a tartrate-precursor combustion route and evaluate them for sub-6 GHz dielectric resonator antennas (DRAs). Structural (XRD, FTIR), microstructural (SEM/TEM), ferroelectric (P–E), thermal-conductivity, and broadband microwave-dielectric (1–15 GHz) characterizations identify x = 0.1 as the composition with the best balance of moderate effective permittivity, low loss, and stable GHz dispersion. A cylindrical DRA is then designed with Sr_0.9_Ba_0.1_TiO_3_ as the matching layer between a Rogers 6010 resonator and a Rogers 4003 feed/ground laminate, and benchmarked against identical-geometry FR-4 and Rogers TMM4 matching layers. Simulated and measured S-parameters confirm efficient impedance matching (|S_11_|_min_ ≈ − 25 dB; VSWR < 2), high realized gain, and stable radiation across 2.25–3.25 GHz, demonstrating that composition-engineered SBTO is a practical option for 5G/early-6G front-ends.

**Supplementary Information:**

The online version contains supplementary material available at 10.1038/s41598-026-61044-1.

## Introduction

The swift 5G roll-out and the 6G horizon have accelerated the need for antenna platforms that can simultaneously achieve miniaturization, broad bandwidth, thermal stability, and high radiation efficiency across sparsely populated microwave spectra^1–5^. Antenna characteristics are closely linked to the electromagnetic (EM) features of their constitutive dielectric materials; permittivity (ε_r_), quality factor (Q_f_), and temperature coefficient of resonant frequency (TCF) dictate size, bandwidth, selectivity, and thermal drift^6–12^. Even though high-ε_r_ materials facilitate strong-field confinement and footprint minimization, they usually sacrifice bandwidth and efficiency, prompting materials strategies to trade off moderate ε_r_ for minimum loss to achieve compact, power-efficient front-ends in future wireless systems^10–12^.

Dielectric resonator antennas (DRAs) are attractive in this regard, as their high radiating efficiency, easily controllable mode, and co-designed material geometry offer the potential to achieve dense, highly efficient, and usable-bandwidth radiators^2,3,6–9,13–21^. They are prone to intrinsic dielectric resonator loss and tunability, whereas perovskite ferroelectric materials offer clear advantages: high, field-responsive permittivity with moderate microwave loss, along with desirable piezoelectric/thermal properties^22,23^. Among them, BaTiO_3_ as well as its solid solutions with Sr (Sr_1−x_Ba_x_TiO_3_, SBTO) permit ε_r_ as well as damping to be composition-controlled through A-site substitution, making SBTO the top contender to be developed as DRA as well as microwave components that demand dispersion-controlled as well as GHz-frequency dielectric loss minimization^24–26^.

Ceramic dielectrics underpin most modern DRA designs because they combine the high ε_r_ (typically 10–100), low microwave loss (tan δ < 10^−3^), and tailorable temperature stability required to miniaturize antennas without sacrificing radiation efficiency^1–12^. Microwave ceramics based on perovskites (e.g., CaTiO_3_–LaAlO_3_^6–12^, BaTiO_3_-derived composites^10–12^, garnets, and complex titanates^6–12^ have repeatedly been demonstrated as resonator cores and substrates for sub-6 GHz and millimeter-wave DRAs. Compared with polymer or glass-reinforced laminates, ceramic DRAs offer superior power handling and thermal stability; relative to single-crystal dielectrics, they offer manufacturability and lower cost. Within this class, Sr_1−x_Ba_x_TiO_3_ is particularly attractive because, unlike fixed-composition titanates, its ε_r_ and ferroelectric-phase boundary can be continuously tuned by the Ba/Sr ratio, opening a composition-engineered design space without altering the host crystal system^22–26^.

Here, we prepare Sr_1−x_Ba_x_TiO_3_ nanoparticles (0.0 ≤ x ≤ 0.4) through a tartrate-precursor Combustion method and conduct full-range structural, microstructural, ferroelectric, thermal, and microwave dielectric measurements to determine an optimized composition of the SBTO material suited to 2–5 GHz operation^2,3^. Based on these data, we conceptually design and construct a cylindrical DRA integrating the best-optimized SBTO material with commercial substrates (Rogers 4003 and Rogers 6010), and we experimentally verify the antenna against full-wave simulation, examining impedance matching, efficiency, gain, and modal fields^2,3,6–9,16,17^. The goal is to show that composition-engineering the SBTO material can be used as a high-quality dielectric core or matching material for DRAs in the 5G/6G-relevant frequency ranges, with a practical trade-off between miniaturization, bandwidth, and radiation loss compared with commercial dielectric materials on their own^10–12,23–26^.

Although Sr_1−x_Ba_x_TiO_3_ has been widely studied as a tunable dielectric and ferroelectric, its deliberate use as a matching layer in a multi-layered DRA co-designed with commercial low- and high-permittivity laminates has not been systematically reported in the sub-6 GHz band. The novelty of the present work is therefore threefold: (i) a single-pot tartrate-precursor combustion route delivers phase-pure SBTO nanoparticles with reproducible composition control across 0 ≤ x ≤ 0.4; (ii) a complete materials-to-device workflow links composition-engineered ε_r_, dielectric loss (tan δ), electrical polarization (P–E), and thermal conductivity (κ) data to an explicit DRA design rule, identifying x = 0.1 as a low-loss, moderate-ε_r_ matching-layer composition; and (iii) the prototype DRA is benchmarked directly against FR-4 and Rogers TMM4 matching options under identical geometry, providing a like-for-like comparison that has been absent from prior SBTO antenna literature.

## Materials and methods

### Preparation of Sr_1−x_Ba_x_TiO_3_ (with x = 0.0, 0.1, 0.2, 0.3, 0.4) nanoparticle samples

By making use of the tartaric precursor technique, Sr_1−x_Ba_x_TiO_3_ (0.0 ≤ x ≤ 0.4) nanoparticles were prepared by the following method: A set of grained reagents of barium nitrate Ba(NO_3_)_2_ (98.5% purity, provided by Qualikems), strontium nitrate Sr(NO_3_)_2_ (≥ 99.0% purity, provided by Techno PharmChem), Titanium dioxide TiO_2_ (≥ 99.0% purity, provided by Techno PharmChem), and tartaric acid C_4_H_6_O_6_ (≥ 99.0% purity, provided by Techno PharmChem), have been weighed and mixed in appropriate ratios with the stoichiometrically calculated molar ratio of 1:1:3 of (Ba and/or Sr) nitrate to TiO_2_ to C_4_H_6_O_6_ respectively after dissolution in distilled water (50 ml for each one), the powders have been stirred for 30 min at room temperature before it’s being heated for 2 h at 80 °C to evaporate the majority of the water. Following that step, the resultant mixture is heated to 120 °C to form a viscous gel, which auto-ignites to yield fine powder nanoparticles. The powder was ground and calcinated for 24 h at 300 °C before sintering for 4 h at 1100 °C. Finally, the powder has been pressed into the desired shape for each measurement^2^.

### Characterization

The ferroelectric samples (Sr_1−x_Ba_x_TiO_3_ (0.0 ≤ x ≤ 0.4)) were characterized by employing X-ray diffraction (XRD) (PANALYTICAL co. Xpert Pro) employing (Cu target, λ = 1.54 Å, 54 kV 40 mA Hollanda) and Fourier transform infrared spectroscopy (FTIR), model Bruker Tensor 27, operating in the 400–4000 cm^−1^ range. The surface microstructure was analyzed using a scanning electron microscope (SEM) model Zeiss EVO 10 (Oberkochen, Germany). The particle size of the Sr_0.9_Ba_0.1_TiO_3_ sample was measured using a transmission electron microscope (TEM) model JEM-2100. Dielectric properties, including the real (ɛ_r_) and imaginary (ɛ_i_) components of permittivity and dielectric loss (tanδ=ɛ_i_/ɛ_r_) components, were extracted from the measured S-parameters employing Rohde & Schwarz ZVA67 vector network analyzer (VNA) along with a dielectric assessment kit (DAK-3.5), and the measured samples were of disk shape with a diameter of 30 mm and a thickness of 5 mm. The sample with x = 0.1 was selected for the design and fabrication of the antenna, as it provided reasonable values of dielectric loss and dielectric constant among all the samples prepared. It should be noted that the ferroelectric properties were measured at room temperature using a RADIANT Precision II Multiferroic Ferroelectric Test System under triangular waveforms with an amplitude of 35 kV/cm and a frequency of 1 Hz. The prepared samples’ thermal conductivity was measured using a Hot Disk Thermal Constants Analyzer.

## Results and discussion

### XRD

**Fig. 1:**
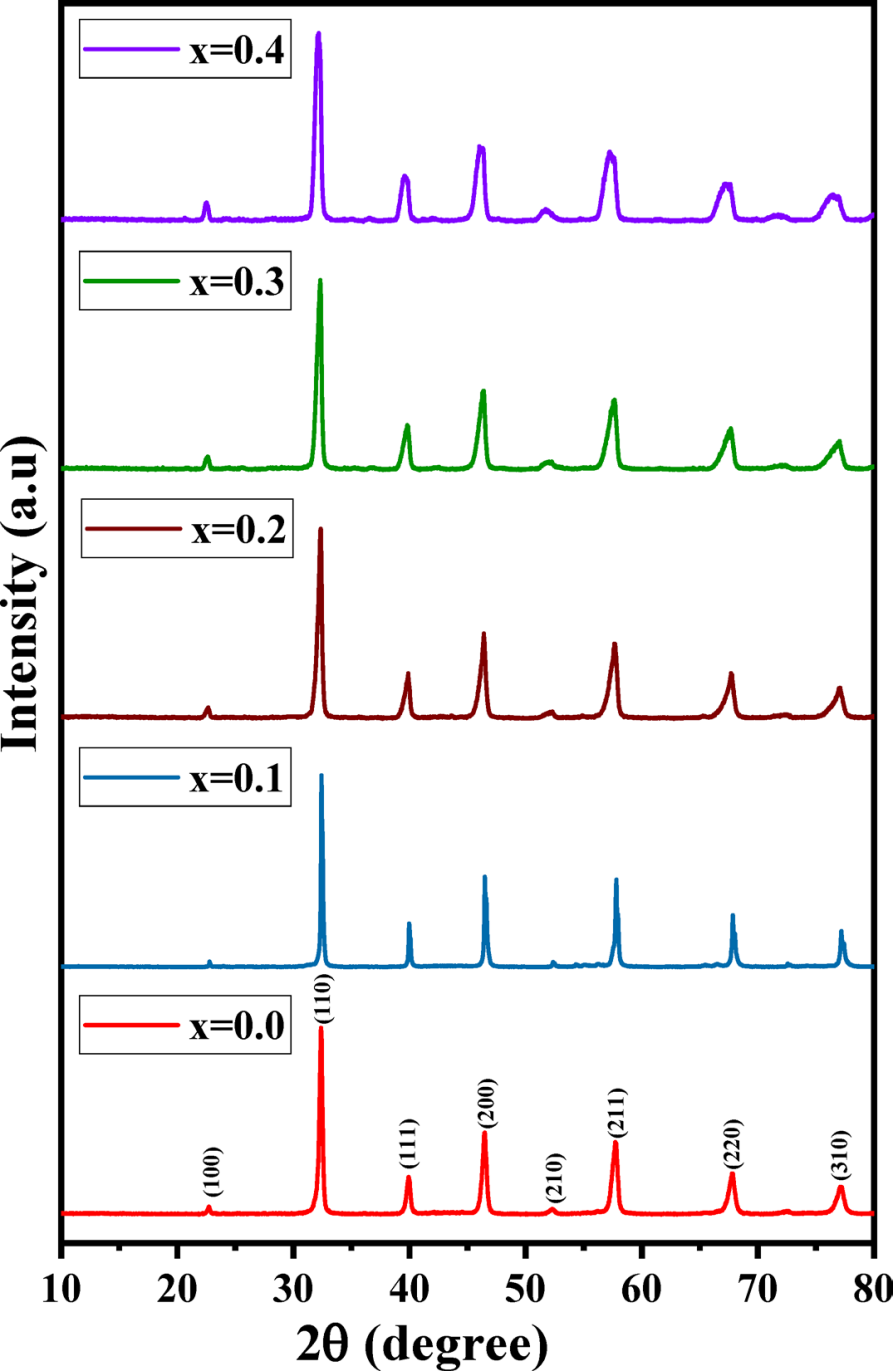
XRD patterns of Sr_1−x_Ba_x_TiO_3_ (0.0 ≤ x ≤ 0.4) samples.

The X-ray diffraction (XRD) patterns for the synthesized ferroelectric nanoparticles Sr_1−x_Ba_x_TiO_3_ with compositions x = 0.0, 0.1, 0.2, 0.3, and 0.4 are shown in Fig. 1. These patterns indicate the formation of a single-phase cubic perovskite structure. Specifically, for the undoped sample SrTiO_3_ (x = 0.0), distinct diffraction peaks were observed at 2θ angles corresponding to crystallographic planes (hkl) at 22.76° (100), 32.37° (110), 39.92° (111), 46.45° (200), 52.18° (210), 57.74° (211), 67.75° (220), and 77.07° (310). These characteristic peaks confirm the formation of the cubic perovskite phase of SrTiO_3_, matching closely with standard reference data (JCPDS card numbers 35–0734 and 79–0176) and are attributed to the Pm-3 m (221) space group^27,28^. Similarly, the XRD patterns for the Ba-doped samples are shown in Fig. 1. The observed diffraction peaks correspond to the aforementioned crystallographic planes, confirming the cubic crystal structure. These peaks are in good agreement with standard data for Sr_0.8_Ba_0.2_TiO_3_ (JCPDS card number 01-080-4496) and Sr_0.6_Ba_0.4_TiO_3_ (JCPDS card number 01-080-4495), both of which are categorized within the space group Pm-3 m (No. 221)^28^.

Although the narrow, sharp diffraction peaks indicate the superior crystalline quality of the synthesized nanocrystals, the minimal peak broadening observed with barium substitution can be attributed to lattice-induced stress, as discussed in detail below. Along with the increased barium content, Fig. 1 also shows a reasonable shift in the diffraction angles (2θ). This is due to variations in d-space directly related to changes in the lattice parameter caused by progressive barium substitution^28^.

Quantitatively, every reflection shifts monotonically toward lower 2θ with increasing Ba content; the most intense (110) peak, in particular, moves from 32.37° at x = 0.0 toward the value reported for the Sr_0.6_Ba_0.4_TiO_3_ reference (JCPDS card 01-080-4495) at x = 0.4. The corresponding d-spacing increase, as predicted by Bragg’s law, is consistent with the substitution of the smaller Sr^2+^ (r_XII_ = 1.44 Å) by the larger Ba^2+^ (r_XII_ = 1.61 Å) on the A-site and follows a Vegard-type compositional dependence.

It is worth noting that the Rietveld XRD patterns are provided in the supporting file. The cubic crystal structure of the space group Pm-3 m was obtained using the VESTA software program, which generated the CIFs for the XRD patterns; these are provided in the supporting file.

### FTIR

**Fig. 2:**
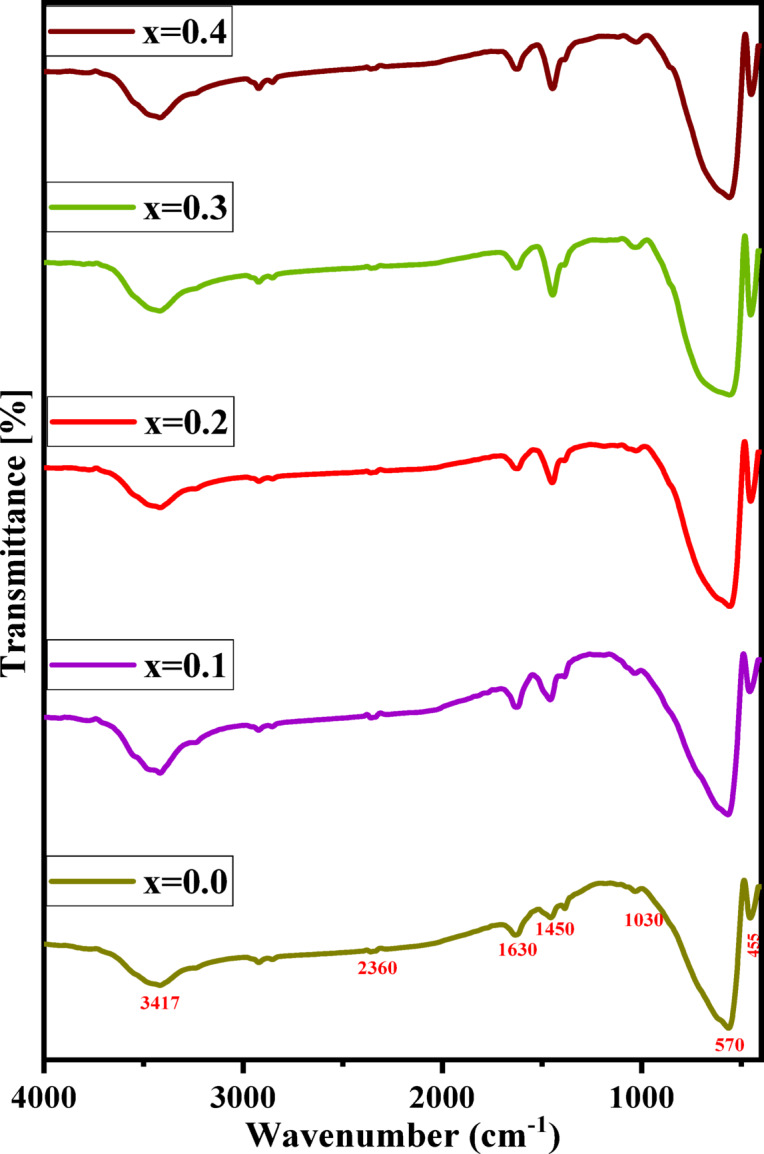
FTIR spectra of Sr_1−x_Ba_x_TiO_3_ (0.0 ≤ x ≤ 0.4) samples.

The FTIR spectra of the synthesized ferroelectric nanoparticles Sr_1−x_Ba_x_TiO_3_ with different compositions (x = 0.0, 0.1, 0.2, 0.3, and 0.4) are given in Fig. 2. The analysis of the spectra clearly shows sharp absorption bands in the region of approximately 455 cm^−1^ ↔ 449 cm^−1^, which indicates the successful development of the cubic-phase BaTiO_3_ Structure. At this specific peak position, the bands refer to the typical vibrational modes of Ti–O bonds. The higher vibrational frequency bands (575 cm^−1^ ↔ 559 cm^−1^) correspond to Ti–O bond-stretching modes, and the lower frequency bands (455 cm^−1^ ↔ 449 cm^−1^) correspond to bending modes. The precise identification of the vibrational modes confirms the development of the perovskite crystalline structure in the prepared samples^29^.

The absorption peak at 1027 ↔ 1036 cm^−1^ is due to the specific C–O–Ti vibrational mode corresponding to tetrabutyltitanate. The 1624 ↔ 1634 cm^−1^ absorption peak corresponds to C=O bond stretching vibrations. Peaks are also observed in the 1446 ↔ 1458 and 2357 ↔ 2360 cm^−1^ bands, corresponding to antisymmetric stretching and bending vibrations of the CO_3_ residue groups (carbonates), respectively. These minor carbonates inevitably form during barium titanate synthesis and exhibit characteristic spectral signatures. Finally, the absorption maximum in the region between 3426 and 3419 cm^−1^ corresponds to the O–H stretching vibrational modes of surface-adsorbed water (H_2_O) molecules^27,28,30^.

Across the series, the Ti–O stretching band shifts monotonically from ≈ 575 cm^−1^ at x = 0.0 to ≈ 559 cm^−1^ at x = 0.4, and the Ti–O bending band from ≈ 455 cm^−1^ to ≈ 449 cm^−1^ over the same compositional range. This red-shift mirrors the lattice expansion observed by XRD (Section  “XRD”): substitution of the smaller Sr^2+^ by the larger Ba^2+^ on the A-site relaxes the TiO_6_ octahedral framework, reducing the effective Ti–O force constant. In contrast, the high-wavenumber bands at 1027–1036, 1446–1458, 1624–1634, and 3419–3426 cm^−1^ show no systematic x-dependence within experimental resolution, confirming that they originate from extrinsic surface-bound species (residual carbonates and adsorbed water) rather than from the perovskite lattice.

### SEM and TEM

**Fig. 3:**
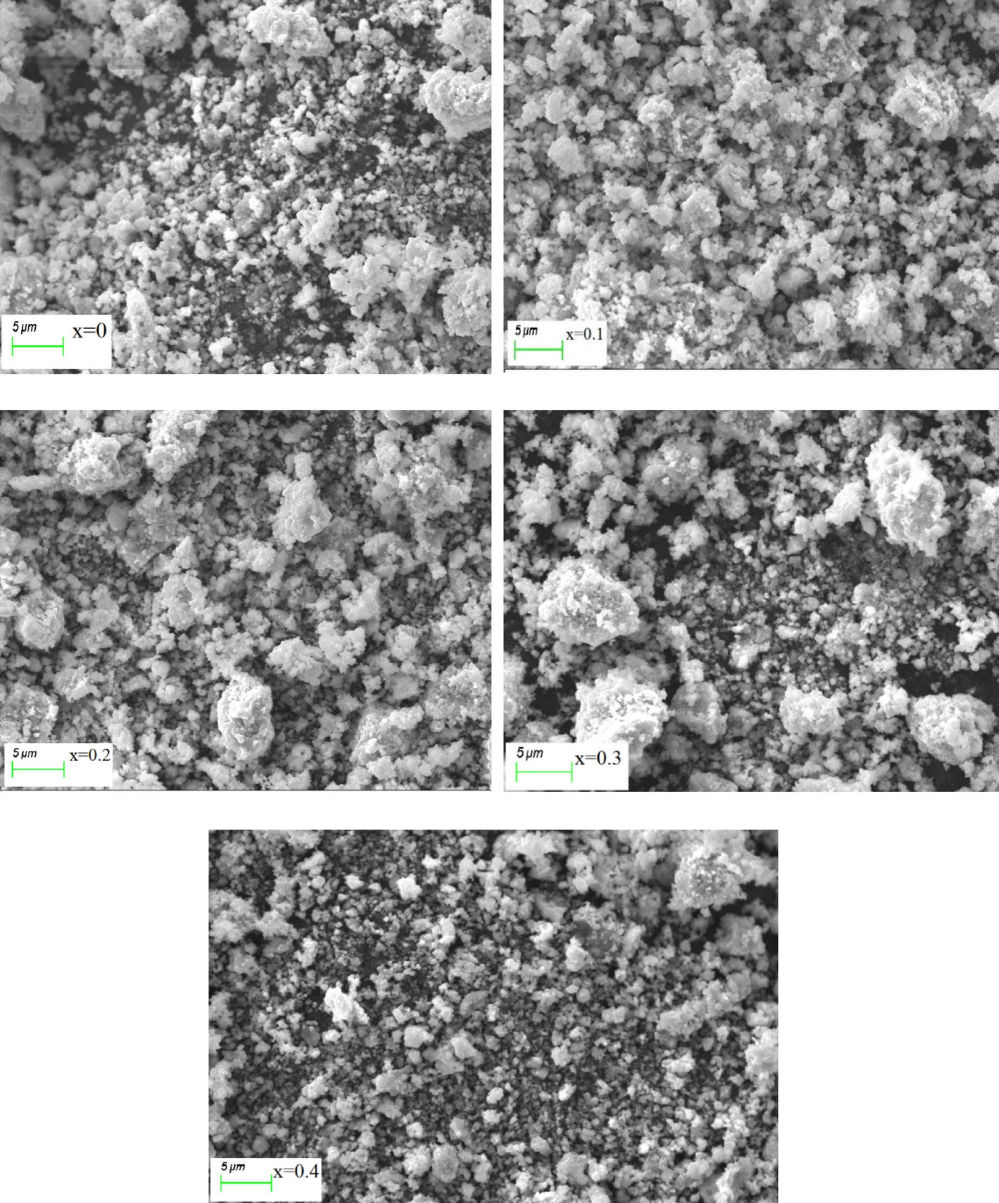
SEM micrographs of Sr_1−x_Ba_x_TiO_3_ (0.0 ≤ x ≤ 0.4).

By SEM analysis, the morphology and surface of Sr_1−x_Ba_x_TiO_3_ (0.0 ≤ x ≤ 0.4) are shown in Fig. 3. This Figure (Fig. 3) shows that the samples are dense across the board, with their nanoparticles forming agglomerates.

**Fig. 4:**
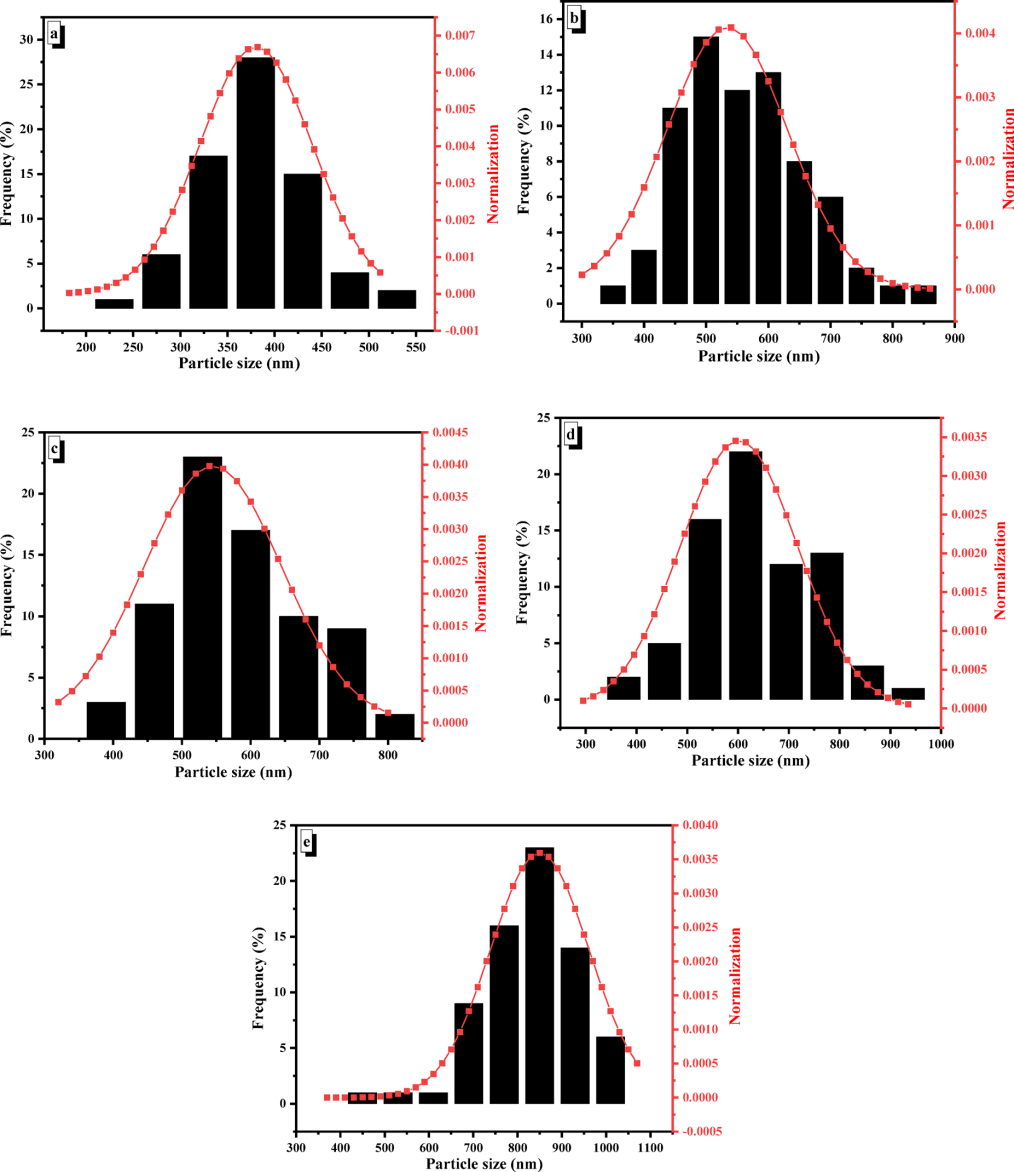
The particle size distribution histogram from SEM of Sr_1−x_Ba_x_TiO_3_ (**a**) x = 0.0 (**b**) x = 0.1, (**c**) x = 0.2, (**d**) x = 0.3, and (**e**) x = 0.4.

The average particle size of all compositions was calculated from SEM images using ImageJ and tabulated in Table 1. Particle surface agglomerations can be inferred from the high standard deviation values reported in Table 1. The histogram of particle size for all the samples is shown in Fig. 4. The histogram exhibits a broad distribution due to the irregular surface of the nanoparticles, which contain random agglomerations^2^.

**Table 1:** Average particle size (nm) and SEM-derived standard deviation for Sr_1−x_Ba_x_TiO_3_ (0.0 ≤ x ≤ 0.4) samples.

Sample	Average particle size (nm) (SEM)	Standard deviation
SrTiO_3_	380	59.58
Sr_0.9_Ba_0.1_TiO_3_	533	97.29
Sr_0.8_Ba_0.2_TiO_3_	544	100.14
Sr_0.7_Ba_0.3_TiO_3_	602	115.34
Sr_0.6_Ba_0.4_TiO_3_	850	108.96

Among the synthesized compositions, x = 0.1 was selected as the representative sample for TEM analysis because the dielectric measurements in “Dielectric properties” section indicate it is the composition down-selected for the antenna prototype. Confirming the nanoscale crystallinity and lattice parameter of this specific composition directly supports the device-side conclusions; the SEM statistics in Table 1 already capture the broader compositional trends in grain size and morphology.

**Fig. 5:**
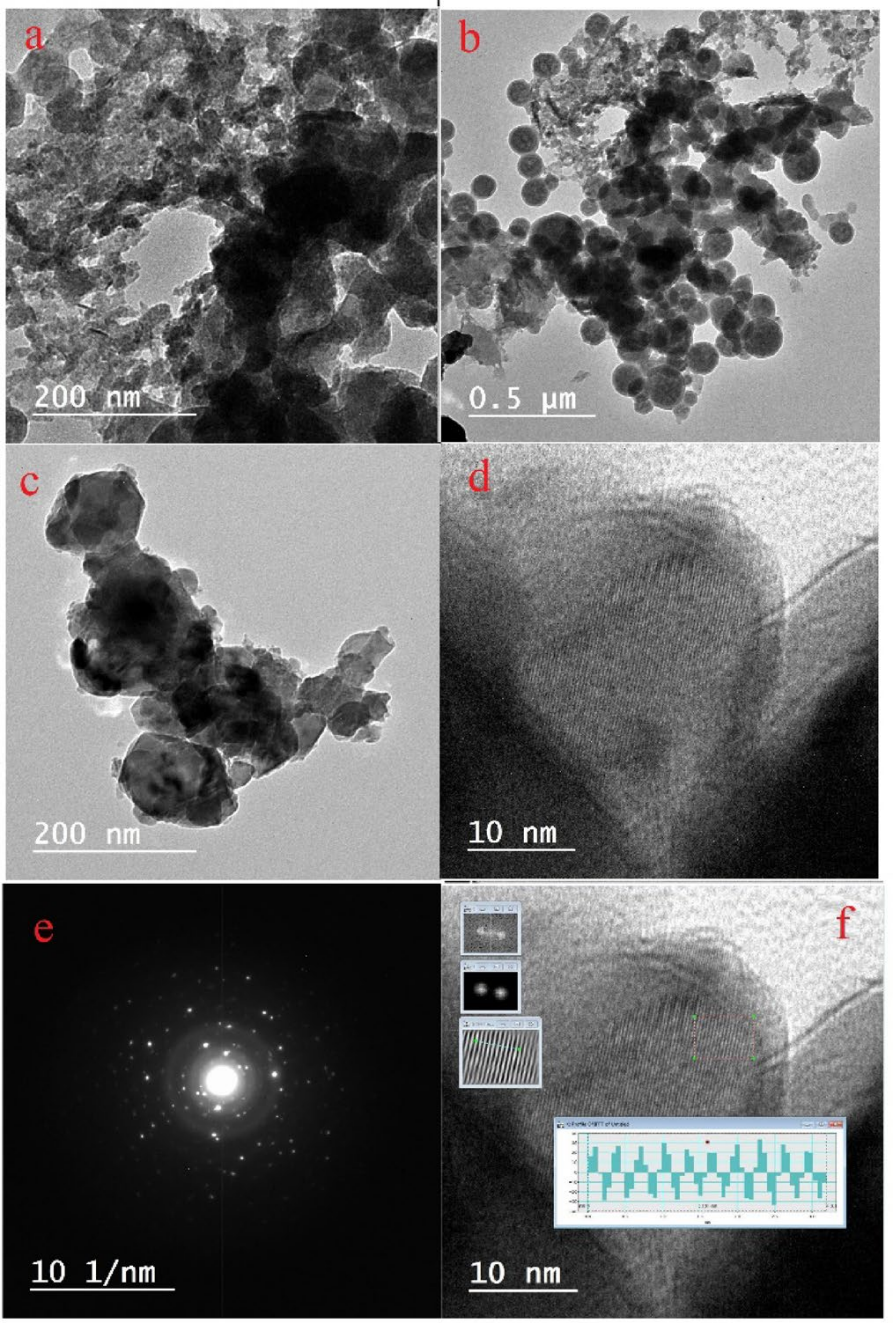
(**a**), (**b**), and (**c**) TEM images of Sr_0.9_Ba_0.1_TiO_3_ in the diffraction mode, (**d**) and (**f**) HRTEM images, and (**e**) SAED pattern.

TEM images of Sr_0.9_Ba_0.1_TiO_3_ nanoparticles are presented in Fig. 5, which shows observable nanoparticle aggregation in the sample. Figure 5d and f show high-resolution transmission electron microscopy (HRTEM), which provides precise information on crystallographic characteristics and lattice order, respectively. Figure 5e shows the selected area electron diffraction (SAED) mode. Quantitative particle size analysis was performed in ImageJ, yielding an average nanoparticle diameter of 34.97 nm. The characteristic concentric ring patterns in the SAED picture substantiate the synthesized crystalline barium titanate nanoparticle^29,31^.

The progressive grain coarsening with increasing Ba content (Table 1) is attributed to accelerated mass transport during the 1100 °C sintering step in Ba-enriched perovskite titanates: the larger Ba^2+^ ion lowers the effective sintering activation energy and raises the A-site cation-diffusion coefficient, so at any given sintering schedule the Ba-rich compositions reach a more advanced stage of normal grain growth, a behaviour previously reported in (Ba, Sr)TiO_3_ ceramics^28,32^.

### Ferroelectric properties

**Fig. 6:**
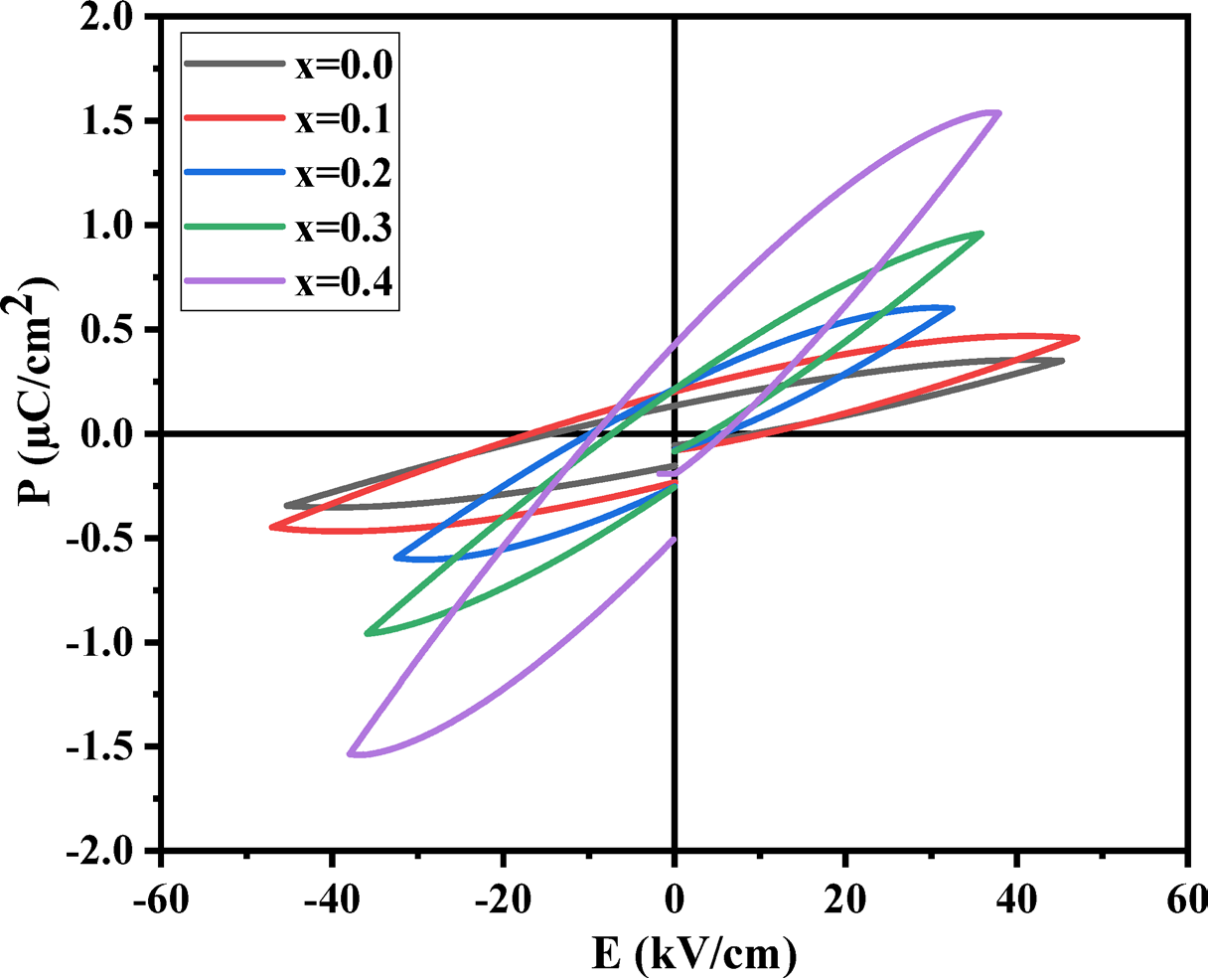
P–E hysteresis loops of Sr_1−x_Ba_x_TiO_3_ (0.0 ≤ x ≤ 0.4).

The ferroelectric hysteresis loops (P–E) for the nanocomposite Sr_1−x_Ba_x_TiO_3_ (where x = 0.0, 0.1, 0.2, 0.3, and 0.4) are shown in Fig. 6.

The observed P–E loops can be analyzed as follows: For an ideal resistor, the current and voltage are in phase, so the P–E loop should be a circle centered at the origin. For an ideal capacitor, the current leads the voltage by 90 degrees; therefore, the charge (the integral of the current with time) is in phase with the voltage. If these two components (ideal resistor and ideal capacitor) are combined in parallel, we get the P–E loop, which is, in effect, a lossy capacitor, where the area within the loop is proportional to the loss tangent of the device, and the slope is proportional to the capacitance^33^.

Returning to the P–E loops, we find that all samples exhibit a lossy capacitor response. Moreover, lossy capacitor behavior samples may be piezoelectric actuators^33^. The loops’ lossy behavior may also indicate a lower electrical resistivity in these samples due to the large leakage current^34^.

Also, Table 2 shows that the saturation polarization (P_s_) and remanent polarization (P_r_) increase with increasing Ba ratio. This increase has primarily been attributed to the ferroelectric phase, which exhibits a low leakage current density, thereby strengthening its ferroelectric nature in Sr_1−x_Ba_x_TiO_3_^35^.

The coercive field (E_c_) values have also decreased sharply with increasing Ba concentration. It may be related to the increase in the ferroelectric Ba phase, resulting in a breakthrough in domain wall motion^36^.

**Table 2:** Parameters obtained from P–E hysteresis loop, P_r_, P_s_, and E_c_ of Sr_1−x_Ba_x_TiO_3_ (where x = 0.0, 0.1, 0.2, 0.3, and 0.4).

Sample	*P*_*r*_ (µC/cm^2^)	*P*_s_ (µC/cm^2^)	E_c_ (kV/cm)
x = 0.0	0.14	0.34	15.46
x = 0.1	0.21	0.45	18.59
x = 0.2	0.22	0.61	7.23
x = 0.3	0.23	0.95	5.81
x = 0.4	0.46	1.53	8.3

### Thermal conductivity properties

#### Effect of Ba concentration on thermal conductivity

**Fig. 7:**
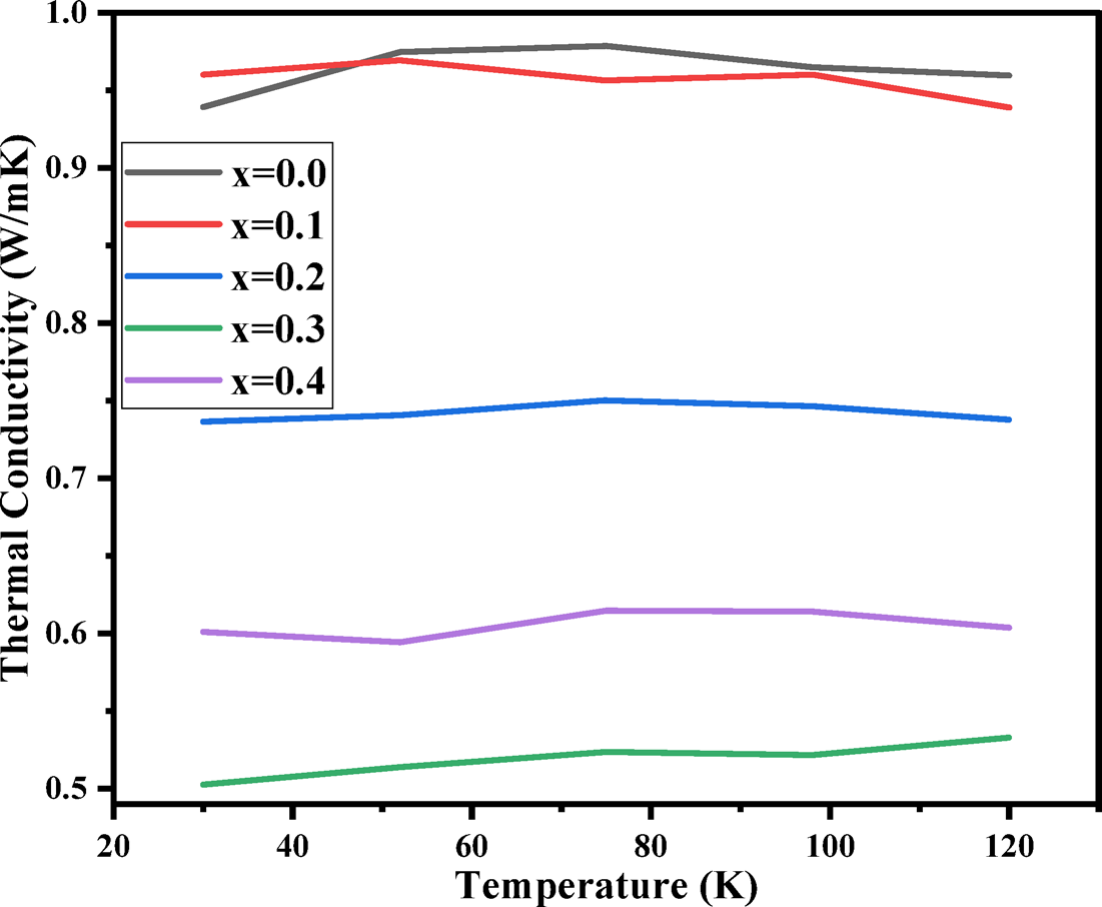
Thermal conductivity vs. temperature of Sr_1−x_Ba_x_TiO_3_ (0.0 ≤ x ≤ 0.4).

Thermal conductivity *κ* as a function of temperature for Sr_1−x_Ba_x_TiO_3_ (x = 0.0–0.4) is shown in Fig. 7. Substitution of Sr with Ba systematically lowers κ over the measured range. The undoped SrTiO_3_ (x = 0.0) sample shows the highest conductivity (peaking at ≈ 1.0 W/m·K), while the Ba-doped samples are progressively lower, falling to ≈ 0.6 W/m·K for x = 0.4, an ≈ 40% reduction at 40% Ba content. The trend is consistent with alloy-type phonon scattering: replacing Sr^2+^ on the A-site with a heavier, larger ion increases lattice mass disorder and impedes heat transport, as described for solid-solution alloy systems^37^. The x = 0.3 composition exhibits the lowest *κ* in the series, slightly below x = 0.4, indicating that phonon scattering is already very intense by 30% Ba and that further substitution gives only a marginal change. The systematic suppression of *κ* with Ba content is unambiguous across the entire measured temperature range.

#### Temperature-dependent thermal conductivity behavior

All compositions show the temperature dependence typical of crystalline insulators. At 30–50 K, *κ* is relatively low for each sample (≈ 0.9 W/m·K for x = 0.0 and ≈ 0.6 W/m·K for x = 0.4). As T rises from 30 K to ≈ 60–80 K, *κ* increases and reaches a broad maximum: pure SrTiO_3_ peaks near 80 K at ≈ 0.95–1.0 W/m·K, and x = 0.4 reaches ≈ 0.62 W/m·K at a comparable temperature. This initial rise reflects the increased thermal population of heat-carrying phonons overcoming the residual scattering from defects and grain boundaries that limits transport at very low temperatures^37^. The Ba-doped samples show a shallower, lower-peak κ; for x = 0.3, κ barely rises between 30 K and 120 K (≈ 0.50 → 0.55 W/m·K), consistent with glass-like thermal transport, in which heavy scattering flattens the temperature dependence^38^.

At higher temperatures (toward 100–120 K), the lower-Ba samples show a mild downturn associated with the onset of Umklapp phonon–phonon scattering, which drives the κ ∝ 1/T behavior expected in the high-T limit^37^. The downturn is less pronounced in the Ba-rich samples because impurity and boundary scattering already dominate, keeping *κ* flat. SrTiO_3_ has a structural cubic-to-tetragonal transition at ≈ 105 K^39^; no sharp anomaly at this temperature is visible in our data, likely because the nanocrystalline microstructure broadens any underlying single-crystal transition. The Ba-substituted samples show no kink in the measured window, indicating that any associated structural or ferroelectric transitions fall outside the 30–120 K range or are diffuse.

#### Phonon scattering mechanisms in Ba-doped SrTiO_3_

The *κ* reduction with Ba doping is attributable to three superimposed scattering channels. First, A-site mass disorder: replacing Sr^2+^ (≈ 87.6 u) with the heavier Ba^2+^ (≈ 137.3 u) creates the point-defect scattering described by Klemens’ alloy-scattering theory; the large mass mismatch (ΔM ≈ 50 u) enhances the scattering rate across a wide range of phonon frequencies. Second, strain-field scattering: Ba^2+^ is larger than Sr^2+^ (r_XII_ = 1.61 Å vs. 1.44 Å)^29^, and the accommodation of the larger ion locally distorts the surrounding TiO_6_ octahedra, producing static strain fields that act as additional phonon-scattering centers. Theoretical analyses indicate that the size-mismatch contribution can be comparable in magnitude to the mass-mismatch contribution in setting the alloy-scattering rate^37^. Third, grain-boundary scattering: the nanostructured ceramics have a dense network of grain boundaries (mean grain size ≈ 530 nm at x = 0.1, Table 1) that truncates the phonon mean free path. Boundary scattering is largely temperature-independent and contributes directly to the flattening of κ(T), particularly in the heavily doped samples whose intrinsic mean free paths are already short.

The combined action of these three channels drives Ba-doped SrTiO_3_ toward a phonon-glass regime: mass disorder, strain fields, and grain boundaries together limit the phonon mean free path so severely that *κ* becomes low and weakly temperature-dependent^38^. Ab initio analyses also indicate that Ba incorporation softens specific polar optical modes and lowers acoustic sound velocities via unit-cell expansion, thereby increasing phonon–phonon scattering rates and further suppressing *κ*^40^. The same combination of mechanisms has been documented in other A-site-disordered perovskite solid solutions^40–43^.

#### Implications for room-temperature DRA operation

The *κ* measurements reported above were acquired between 30 K and 120 K. This temperature window is sufficient to resolve the composition-driven phonon-scattering trend that underpins the materials-down-selection logic of the paper. However, it does not directly span the room-temperature regime in which the proposed DRA operates. We therefore address the implications for room-temperature operation explicitly here.

For dense polycrystalline SrTiO_3_ and BaTiO_3_ ceramics, well-established room-temperature thermal conductivities of ≈ 6–10 W/m·K and ≈ 2–3 W/m·K, respectively, are reported in the literature^39,40^. Our measured values in the 30–120 K window already lie an order of magnitude below the corresponding bulk single-crystal values, due to the combined contributions of alloy, strain-field, and grain-boundary scattering in the nanostructured compact (Section “Phonon scattering mechanisms in Ba-doped SrTiO_3_”). Extrapolation along the Umklapp 1/T tail visible at the high-T end of Fig. 7, anchored to the published bulk-ceramic values for the SrTiO_3_ and BaTiO_3_ end-members, places the room-temperature *κ* of Sr_0.9_Ba_0.1_TiO_3_ in the 1–3 W/m·K window, modest, but not pathologically low.

**Fig. 8:**
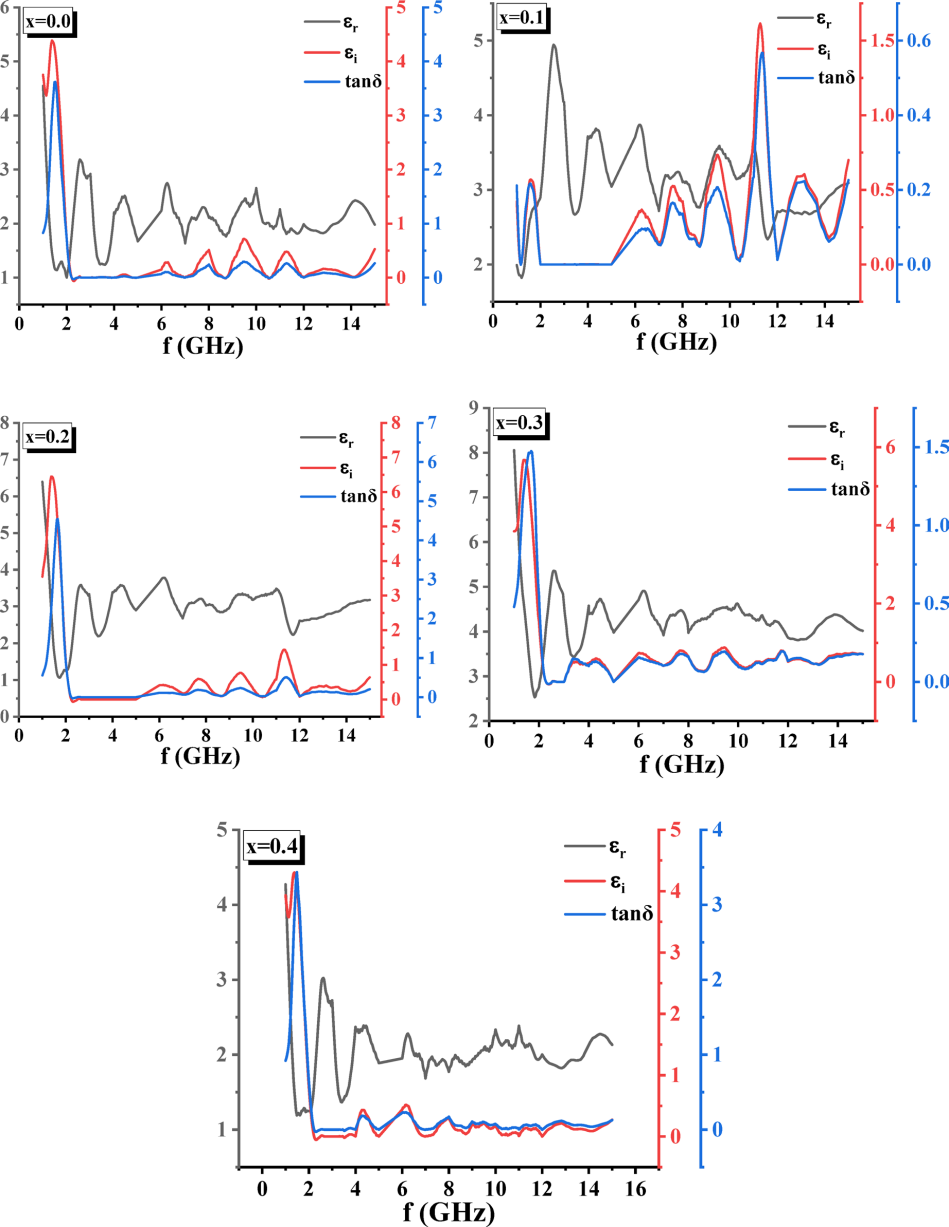

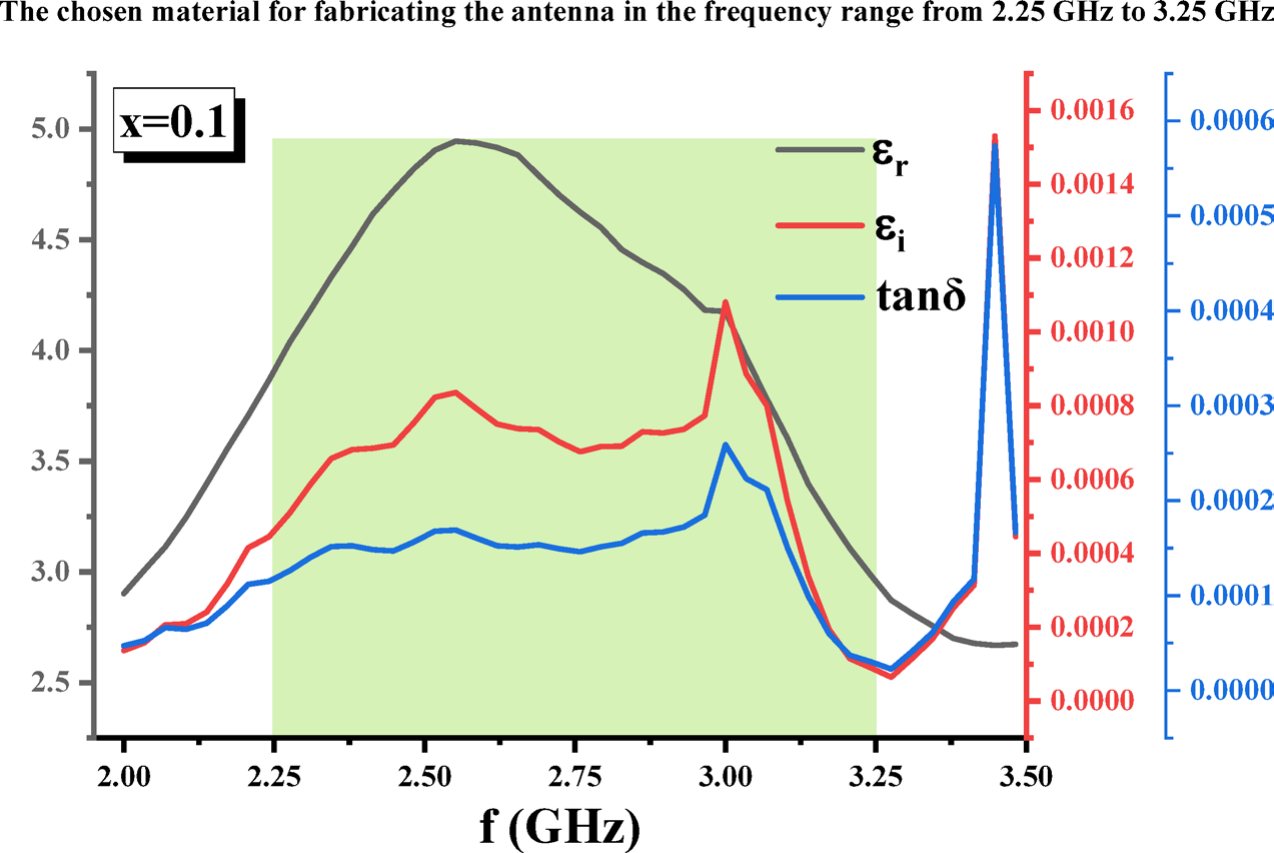
Real (ɛ_r_) and imaginary (ɛ_i_) components of permittivity and dielectric loss (tan δ = ɛ_i_/ɛ_r_) vs. frequency (f) for the prepared Sr_1−x_Ba_x_TiO_3_ (0.0 ≤ x ≤ 0.4).

A simple steady-state estimate bounds the consequences of this modest *κ* for antenna operation. At sub-6 GHz handset and small-cell input powers (typically ≤ 1 W) and with the band-averaged tan δ measured for Sr_0.9_Ba_0.1_TiO_3_ across 2.25–3.25 GHz (Section “Dielectric properties”, Fig. 8), the dielectric power dissipated in the matching disk (radius 15 mm, thickness 5 mm; volume V ≈ 3.5 cm^3^) is bounded above by a few tens of milliwatts even under continuous-wave excitation. With *κ* ≈ 1–3 W/m·K and direct thermal contact to the metalized ground plane on one face, a one-dimensional steady-state estimate (ΔT ≈ Q·t / (κ·A), with Q the dissipated power, t = 5 mm, and A = π·r^2^) yields a temperature rise on the order of a fraction of a kelvin to a few kelvin. This is well below any threshold for dielectric drift, depolarisation, or mechanical failure in the perovskite-titanate family, and the antenna response is therefore not expected to detune appreciably from self-heating at these operating powers.

For higher RF powers or higher duty cycles, the picture changes quantitatively but not qualitatively: volumetric dissipation scales linearly with input power and with tan δ, while the steady-state ΔT scales inversely with *κ*. Adequate thermal margin can be preserved by maximizing the contact area between the matching disk and the metalized ground plane, by bonding the resonator stack to a thermally conductive carrier, or by reducing tan δ through optimized sintering. The mechanical-retention bonding scheme adopted in our prototype (Section “Design analysis”) was deliberately chosen to preserve the thermal pathway from the disk to the ground plane without inserting a low-thermal-conductivity adhesive in the heat-flow path.

Direct measurement of the steady-state temperature rise under sustained RF excitation at the operating band requires a calibrated thermal-imaging or thermocouple setup in an anechoic chamber and is outside the scope of the present materials-focused study. We identify it as the natural next step and as a prerequisite for any application requiring a quantitative thermal-stability margin under continuous-wave operation.

### Dielectric properties

#### Dielectric behavior of Sr_1−x_Ba_x_TiO_3_ nanoparticles in the microwave range (Frequency-dependent permittivity and loss trends (1–15 GHz))

Broadband measurements of Sr_1−x_Ba_x_TiO_3_ (x = 0.0–0.4) reveal characteristic dispersion in the complex permittivity over 1–15 GHz. In all compositions, as shown in Fig. 8, the real part of the permittivity (ε_r_) decreases gradually with increasing frequency^44^. This decline is expected because fewer polarization mechanisms can follow the alternating field at higher frequencies, leading to a decrease in the stored dielectric response. For example, in perovskite titanates, a reduction of ε_r_ by several tens of percent from low MHz to multi-GHz frequencies is commonly observed as dipolar and ionic polarizations begin to lag behind the field^44,45^. No sharp resonance is observed up to 15 GHz, indicating that the dominant polarization mechanisms (e.g., lattice vibrations) have natural frequencies well above this band (in the far infrared). Therefore, the 1–15 GHz response is a depolarization tail rather than a distinct resonance. The imaginary part of permittivity (ε_i_) and the dielectric loss tangent (tan δ = ε_i_/ε_r_) tend to increase with frequency for these ceramics^44^. At low GHz frequencies, Sr_1−x_Ba_x_TiO_3_ is in a low-loss regime (tan δ on the order of 10^−2^ or less), but as the frequency approaches the upper end of the measurement range, more energy is dissipated per cycle. This yields a rising tan δ, especially pronounced in samples closer to ferroelectric resonance conditions (higher Ba content)^44^. In the measured 1–15 GHz band, the loss spectra exhibit broad peaks or plateaus rather than a flat response, indicating a wide distribution of relaxation processes. Indeed, ferroelectric perovskites, such as Sr_1−x_Ba_x_TiO_3_, exhibit frequency-dependent dielectric losses that increase with GHz frequency due to the onset of polar-optic mode damping^44^. There is no sudden loss spike in our measurements (other than small experimental artifacts), but at each composition, an otherwise gentle upward slope is observed in ε_i_(f) and in the material’s tangent loss δ(f). This agrees with earlier broadband work on Sr_1−x_Ba_x_TiO_3_, which showed a “strong increase in losses and dispersion with increasing frequency” as one approached microwave frequencies^44^.

The dispersion of ε_r_ across 2–4 GHz visible in Fig. 8 for x = 0.1 has both an intrinsic and an extrinsic component. Intrinsically, SBTO compositions close to the paraelectric/ferroelectric boundary exhibit soft-mode-driven dispersion augmented by Maxwell–Wagner interfacial polarisation and slow polar-nanoregion relaxations, with magnitude consistent with prior reports on related titanates^47–50^. Extrinsically, methodology stability was verified by three independent sweeps with probe re-contacting and full short/open/load recalibration between sweeps; the run-to-run scatter on ε_r_ at 3 GHz remained below ≈ 3% for x = 0.1, well below the dispersion observed in Fig. 8. The sample-to-sample variation is therefore physical, and the 3% envelope is propagated into the antenna sensitivity analysis of “Design analysis”.

Each composition exhibits the same qualitative trend (ε_r_ ↓, ε_i_ ↑ with increasing frequency), but the scale of these variations depends on the Ba content. The higher Ba compositions exhibit stronger dispersion: e.g., contrasting ε_r_ at 2 GHz and 15 GHz, the decrease in permittivity is larger for x = 0.3–0.4 than for x = 0.0–0.1. This is because the Ba-rich Sr_1−x_Ba_x_TiO_3_ is closer to the polar phase and includes slower polarization modes (soft phonons, domain wall motions) that already start to “freeze out” in the microwave region^44^. In contrast, the SrTiO_3_ end member (x = 0) is an incipient ferroelectric with a high-frequency soft mode; its ε_r_ is relatively flat across 1–15 GHz (only a slight decrease), and tan δ remains extremely low (on the order of 10^−3^–10^−2^) throughout the band. Thus, increasing frequency uniformly tends to lower the dielectric constant and raise the loss tangent for all samples, but Ba-rich samples disperse more strongly, while Sr-rich samples maintain more frequency-stable dielectric properties^44^.

#### Influence of Ba substitution (x) on dielectric response

Substituting Ba^2+^ for Sr^2+^ in Sr_1−x_Ba_x_TiO_3_ dramatically alters the dielectric behavior. At quasi-static frequencies (e.g., 1 kHz), adding Ba is well known to increase the permittivity by shifting the material closer to ferroelectric order (BaTiO_3_ has ε_r_ ∼10^4^ near its Curie point, whereas SrTiO_3_ is a quantum paraelectric with lower ε_r_ at room temperature)^45^. In the microwave regime, we observe the same qualitative trend: ε_r_ (0–GHz) increa**ses** with Ba content, but the absolute values are lower than those at low frequencies due to dispersion. For instance, it has been reported that at 1 GHz, the bulk dielectric constant of Ba_0.25_Sr_0.75_TiO_3_ was approximately 1600, and that of Ba_0.1_Sr_0.9_TiO_3_ was approximately 200^45^. This titanic decrease with increasing Sr (and the reduction in the Ba fraction) indicates how highly sensitive the permittivity is to composition. In the region of our compositions (0 ≤ x ≤ 0.4), the low-frequency permittivity decreases with decreasing x; the x = 0.4 sample shows the maximum ε_r_, and the x = 0.0 sample shows the lowest. The Ba-rich ceramics with compositions close to the ferroelectric phase transition (high lattice polarizability) accordingly carry a higher baseline dielectric constant than SrTiO_3_^45^.

Nevertheless, increased Ba content also leads to a significant increase in dielectric loss. We observed that tan δ increases substantially at microwave frequencies as x increases from 0 to 0.4. This agrees with the literature: e.g., Ioachim et al. reported that increasing x from 0.25 to 0.90 in Ba_1−x_Sr_x_TiO_3_ (i.e., reducing Ba content from 75% to 10%) caused the microwave loss tangent to fall sharply from approximately 12% to below 2%^45^. The Ba-rich composition closer to BaTiO_3_ (x ≈ 0) had tan δ ≈ 0.12 at 1 GHz, while the Sr-rich composition had tan δ ≈ 0.02 at the same frequency. This aligns with our observations that Ba substitution tends to increase tan δ, an undesirable but unavoidable trade-off. BaTiO_3_-based perovskites inherently exhibit higher losses at GHz frequencies due to their stronger polar soft modes and domain-wall contributions^46^. In comparison, SrTiO_3_ (x = 0) is a paraelectric with comparatively low microwave loss. Generally, it is reported that “Sr_1−x_Ba_x_TiO_3_ material possesses high dielectric loss in the microwave region,” particularly on the Ba-rich side^46^. Our measured ε_i_ for x = 0.3–0.4 is much higher than for x = 0.0–0.1, indicating larger dielectric damping with increasing Ba content.

#### Polarization mechanisms and microstructural effects

Lattice distortion and phase transition characteristics significantly influence the dielectric properties of Sr_1−x_Ba_x_TiO_3_. The cubic perovskite lattice of SrTiO_3_ at room temperature (paraelectric) becomes tetragonal (ferroelectric) in BaTiO_3_. Introducing Ba into SrTiO_3_ gradually increases the unit cell volume and enhances the off-centre Ti displacement, thereby increasing the Curie temperature (T_c_)^46^. Even for x = 0.4 (40% Ba), the material remains in the paraelectric phase at 25 °C (T_c_ remains below room temperature), but the local polar distortions become considerably stronger than in pristine SrTiO_3_. XRD analysis shows that the average structure remains cubic for the modest Ba content, though the lattice is disturbed by size mismatch and the onset of incipient polar order^46,47^. This lattice distortion increases the static permittivity and adds anharmonicity to the soft optical phonon mode. Consequently, the soft mode frequency (the inherent vibration frequency of the polar lattice mode) reduces with Ba substitution^44^. A reduced soft-mode frequency directly implies that the dielectric response will be more dispersive in the GHz regime, because the dielectric constant becomes nondeterministic at frequencies that represent a larger fraction of the soft-mode frequency^44^. Essentially, lattice distortion induced by Ba drives the material toward ferroelectric instability with higher ε_r_ but a “softer” lattice that is more readily damped at microwave frequencies.

Relaxation mechanisms driven by dipolar dynamics are evident in the measured dielectric spectra, further explaining the observed frequency and loss behavior. In an ideally symmetric paraelectric such as SrTiO_3_, polarization mainly stems from ionic displacements that resonate in the far-IR (tens of THz) with negligible loss at microwave frequencies. However, in Ba-substituted compositions, particularly for x ≈ 0.2–0.4, polar nanoregions or relaxing dipoles may exist on much slower time scales^44^. These may correspond to embryonic ferroelectric domains or local Ba-enriched areas with higher T_c_. The presence of such polar regions leads to Debye-like relaxation behavior in the GHz range: as the measurement frequency passes through the characteristic relaxation frequency of these dipoles, a peak in εi (and tan δ) appears, accompanied by a faster decrease in ε_r_. This mechanism likely underlies the notable loss peaks observed around a few GHz in the x = 0.2–0.4 samples. It is supported by prior analyses showing that a broad distribution of relaxation times (Cole–Cole type dispersion) exists in Sr_1−x_Ba_x_TiO_3_ near its Curie temperature, causing losses to extend over several decades of frequency, including the microwave band^44^. In our example, variations in Ba/Sr and strain in the polycrystalline microstructure may broaden the relaxation, smoothing it into a gradual frequency dependence rather than a sharp Debye peak. However, the impact is irreversible: the higher tan δ of Ba-rich is attributed to relaxations in domain or dipolar states due to energy absorption by vibrating domain walls and nano-dipoles. Such a loss, characteristic of the material’s ferroelectric or polar phase precursor^32^, has been observed.

Extrinsic mechanisms and grain boundaries also contribute to dielectric properties, particularly in ceramic materials. Sr_1−x_Ba_x_TiO_3_ samples are polycrystalline, with potential grain-interface barriers that can trap moving charges or prevent domain-wall motion. These inhomogeneities result in Maxwell–Wagner polarization at low frequencies, essentially a buildup of charge at grain interfaces that increases the low-frequency permittivity but can also generate a relaxation loss at a characteristic frequency linked to the RC time constant of the grain boundary^48^. If the grain boundaries in our samples are resistive, they could contribute to the dielectric loss in the GHz range by interfacial relaxation. Indeed, extrinsic losses in ferroelectric materials are often attributed to charged defects and grain boundaries, which add a microwave loss term to the intrinsic lattice damping^32^. Moreover, grain boundaries pin ferroelectric domain walls. Pinned domain walls respond less to AC fields (reducing ε_r_ somewhat) but can still oscillate locally and dissipate energy, contributing to tan δ. The net effect of grain boundaries usually lowers the effective permittivity (since not all of the grain interior polarization is fully utilized) and introduces additional loss pathways. High-density, large-grain ceramics tend to have lower loss tangents, whereas finer-grained or porous samples show higher losses^49^. In our discussion, we assume the samples are well-sintered; even so, some microwave loss can be ascribed to extrinsic mechanisms (domain wall friction, defect dipole relaxations, and space charge at interfaces). In addition to the intrinsic damping of the soft mode described earlier, these extrinsic factors help explain why the measured tan δ (e.g., a few percent) is higher than one would expect for an ideal cystal. Methods such as acceptor doping (e.g., with Mn^2+^) are known to pin domain walls and significantly reduce Sr_1−x_Ba_x_TiO_3_ microwave losses (Q improvement)^45^, underscoring the importance of controlling these mechanisms to broaden the material’s performance.

The combination of (i) soft-mode-driven intrinsic dispersion, **(ii)** Maxwell–Wagner interfacial polarisation at grain boundaries, and **(iii)** slow relaxations associated with polar nanoregions accounts for the structured, non-monotonic (‘wavy’) ε_r_(f) and ε_i_(f) trends visible in Fig. 8 for the higher-x compositions. Each mechanism operates in an overlapping but distinct frequency window and increases in amplitude with Ba content, which explains why the x = 0.0 sample shows nearly monotonic dispersion, while the x ≥ 0.2 samples exhibit pronounced features. This three-mechanism picture is in agreement with recent broadband studies of compositionally related perovskite and titanate systems^50–52^.

#### Selection of Sr_0.9_Ba_0.1_TiO_3_ for 2–5 GHz Antenna Design

Due to its well-balanced dielectric characteristics, the Sr_0.9_Ba_0.1_TiO_3_ composition (x = 0.1) emerged as the best material for an antenna in a microwave device operating at 2–5 GHz. This material has a medium permittivity at the GHz frequencies – neither too small nor too large. A medium ε_r_ (of the order of several tens) is preferable for a dielectric resonator antenna substrate as it minimizes device volume while preserving bandwidth and impedance match. If ε_r_ is excessively large (as in Ba-rich Sr_1−x_Ba_x_TiO_3_, which may exceed 100 or even 1000 near the Curie point^45^), an antenna becomes highly electrically small and narrowband, potentially reducing its radiation efficiency. In contrast, pure SrTiO_3_ (x = 0) has a relatively low permittivity in the microwave range (in our measurements, effective ε_r_ was only a few units, partly due to measurement geometry), which would result in a larger antenna size. The x = 0.1 sample provides an ideal balance: its dielectric constant in the 2–5 GHz band is high enough to miniaturize the antenna (much higher than typical polymer substrates, ε_r_ ~ 2–4) but not so high as to make the antenna excessively small or overly sensitive. Sr_0.9_Ba_0.1_TiO_3_ demonstrates a low dielectric loss tangent in the 2–5 GHz range. Our measurements and earlier reports indicate that tan δ for x ≈ 0.1 is below 0.01 at microwave frequencies^45^. This is essential for antenna applications to prevent significant RF power from being lost in the substrate as heat and to maintain radiation efficiency.

A higher x composition (e.g., x = 0.4) could possess several percent of tan δ, dampening signals and antenna gain. The x = 0.1 composition, which is well within the paraelectric regime at room temperature, avoids the severe damping features associated with ferroelectric domain dynamics. It remains in a low-loss dielectric state with < 1–2% losses in the 2–5 GHz frequency band, similar to many typical microwave ceramics^45^. Indeed, scientists reported that some Sr_1−x_Ba_x_TiO_3_ ceramics in their paraelectric phase are very suitable for RF devices with “losses below 1% between 2 and 3 GHz” and a reasonable dielectric constant^53^. Our selected Sr_0.9_Ba_0.1_TiO_3_ composition optimizes this favorable regime.

Other practical reasons in favor of Sr_0.9_Ba_0.1_TiO_3_ antennas are: its permittivity is modest and highly frequency-stable in the 2–5 GHz band (flat dispersion), which facilitates antenna design; the designer can assume an approximately constant εr in this band. Further, without any phase transition, the composition would also be free of a low-temperature permittivity coefficient, so the antenna’s performance would not vary drastically with fluctuations in surrounding temperature. The modest ε_r_, combined with low tan δ, directly yields an appropriate material figure of merit for microwave applications that both require miniaturization and high efficiency^53^. By employing the x = 0.1 material as a dielectric resonator or substrate, the antenna can achieve a reasonable balance between size, bandwidth, and gain. By comparison, an x = 0.2–0.4 material would potentially be smaller (higher ε_r_) but higher loss and narrower bandwidth due to the higher-Q resonance (since high-εr dielectric resonator antennas (DRAs) inherently have higher Q and lower fractional bandwidth^53^). Conversely, x = 0 provides low loss but insufficient permittivity enhancement to warrant employment over conventional low-loss substrates.

**Fig. 9:**
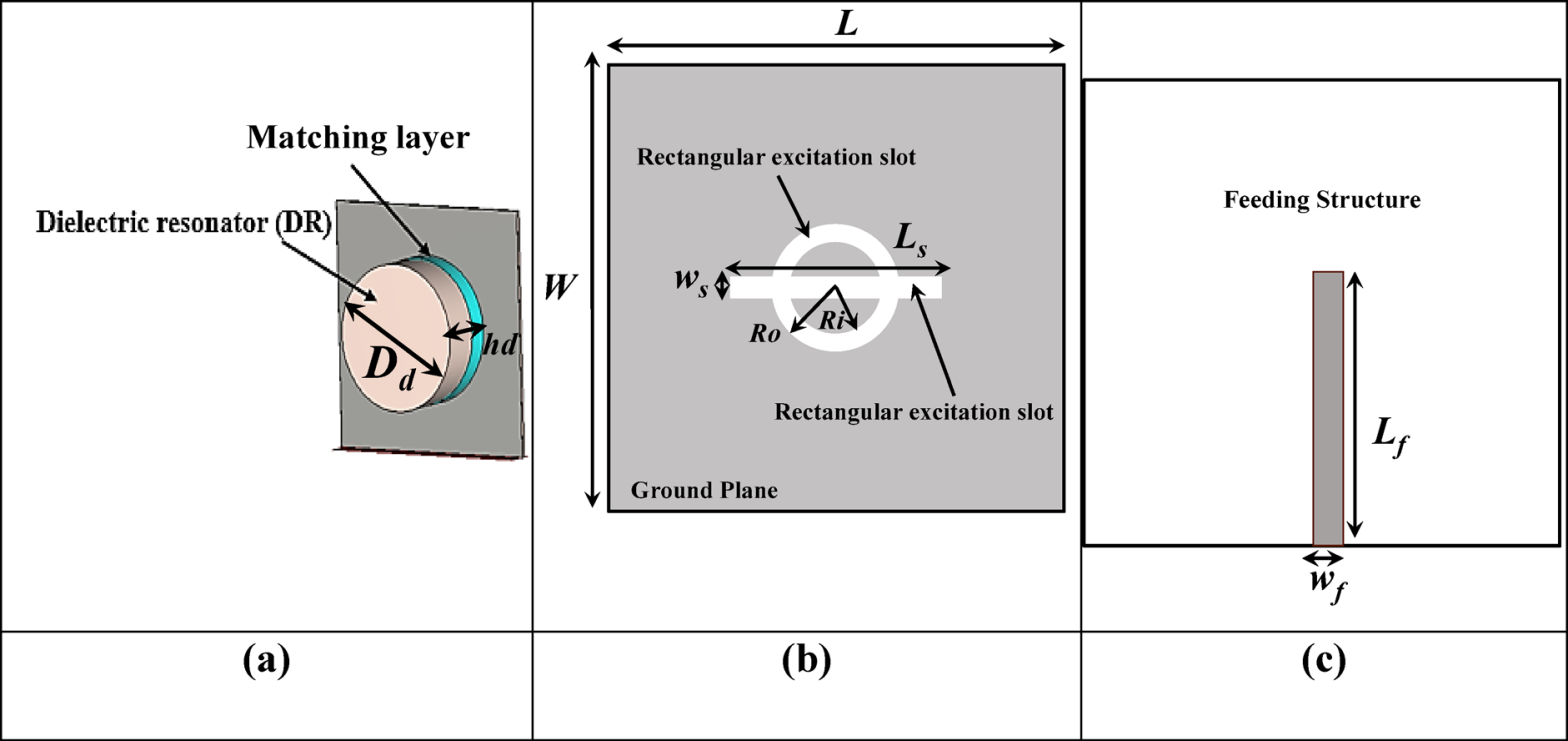
Antenna geometry: (**a**) Full antenna structure, (**b**) Ground plane, including excitation slot, and (**c**) Feeding line.

The effective ε_r_ ≈ 4.3 obtained on our pressed-and-sintered nanoparticle disk is lower than the ε_r_ ≈ 200 reported for fully dense bulk Ba_0.1_Sr_0.9_TiO_3_ at 1 GHz^45^. The reduction is consistent with the residual porosity and the large grain-boundary fraction of the nanoparticle compact, as well as with the effective-medium dilution of the dielectric response, well documented for porous ferroelectric titanates. For antenna design, what matters is the effective ε_r_ of the actual fabricated disk; the measured value places Sr_0.9_Ba_0.1_TiO_3_ in a useful intermediate range between standard low-loss laminates (Rogers 4003, ε_r_ = 3.55) and high-ε_r_ microwave ceramics (Rogers 6010, ε_r_ = 10.2), enabling its use as the matching layer in Fig. 9.

### Antenna design and characteristics

#### Design geometry

The geometric design of the cylindrical dielectric resonator (CDR) is a critical factor in its performance, as it directly influences its EM behavior and resonance characteristics. As shown in Fig. 9, the resonator is constructed using a multi-layered approach, with each layer serving a specific purpose in achieving the desired functionality. The substrate comprises Rogers 4003 with a thickness of $\:\mathrm{h}\:=\:$0.813 mm and total dimensions of 50 × 50 mm^2^. Being slightly lossy in the dielectric, this substrate provides a low-loss base. The substrate’s back is formed of a Sr_0.9_Ba_0.1_TiO_3_ sample with a thickness of $\:{\mathrm{h}}_{\mathrm{m}}=\:\:$5 mm and a radius of 15 mm. A 10 mm thick Rogers 6010 is deposited atop the Sr_0.9_Ba_0.1_TiO_3_ sample. The Rogers 6010, with its high dielectric constant, is selected because it helps localize the EM fields within the resonator’s material structure. By integrating both materials, the dielectric properties of the resonator can be tailored to achieve optimal performance within the target frequency band. It should be noted that each material in the proposed antenna structure was selected for a specific electromagnetic and practical function. Rogers 4003 is used as the bottom microwave substrate because it provides a stable, low-loss platform for the microstrip feedline and ground-plane patterning while maintaining good manufacturability at sub-6 GHz frequencies. Rogers 6010 is used as the main cylindrical dielectric resonator layer because its relatively high dielectric constant helps confine the electromagnetic field inside the resonator and supports compact DRA operation. The Sr_0.9_Ba_0.1_TiO_3_ ceramic layer is introduced as the investigated composition-engineered dielectric material. Its role is to evaluate the applicability of the optimized SBTO ceramic as an intermediate/matching dielectric layer in the DRA stack, where it can influence the effective dielectric loading, field distribution, and impedance matching between the feed and the resonator.

The geometry of the proposed DRA is illustrated in Fig. 9, and the complete set of optimized dimensional parameters is summarized in Table 3. The antenna is excited through a microstrip feeding line having a width of (W_f_ = 3.5) mm and a length of (L_f_ = 30) mm. The defective ground structure consists of a circular ring slot intersected by a rectangular excitation slot, as shown in Fig. 9b. The circular ring slot has an outer radius of (R_o_ = 7.5) mm and an inner radius of (R_i_ = 5.5) mm, whereas the rectangular slot has a width of (W_s_ = 2.5) mm and a length of (L_s_ = 24) mm. This combined slot configuration is employed to control the electromagnetic coupling between the feeding line and the cylindrical dielectric resonator and to achieve suitable impedance matching over the intended operating band.

**Fig. 10:**
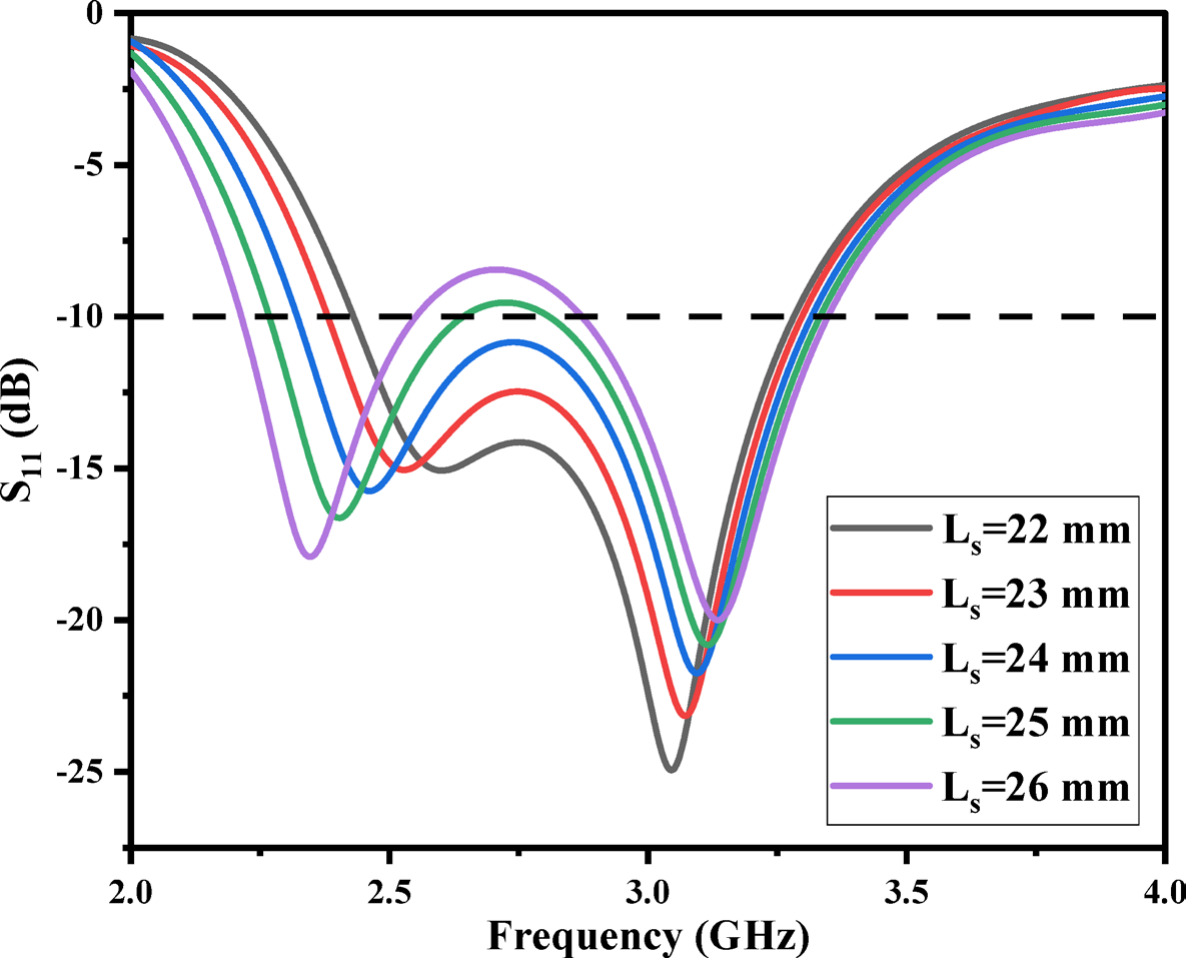
Simulated parametric sweep on the slot length (L_s_).

To investigate the influence of defect dimensions on the antenna response, parametric analyses were performed on the rectangular-slot length (L_s_) and the outer radius of the circular ring slot (R_o_). In contrast, all other antenna dimensions and material properties were held constant. Figure 10 presents the simulated reflection coefficient for (L_s_) values ranging from 22 to 26 mm. It can be observed that increasing (L_s_) progressively shifts the lower-frequency resonance toward lower frequencies, indicating that the rectangular excitation slot strongly influences the electrical coupling path associated with the lower resonant region. In contrast, the upper resonance remains near 3.1 GHz, although its matching level changes slightly with (L_s_). The selected value of (L_s_ = 24) mm provides satisfactory matching at both resonant regions and avoids excessive shifting of the lower resonance.

**Fig. 11:**
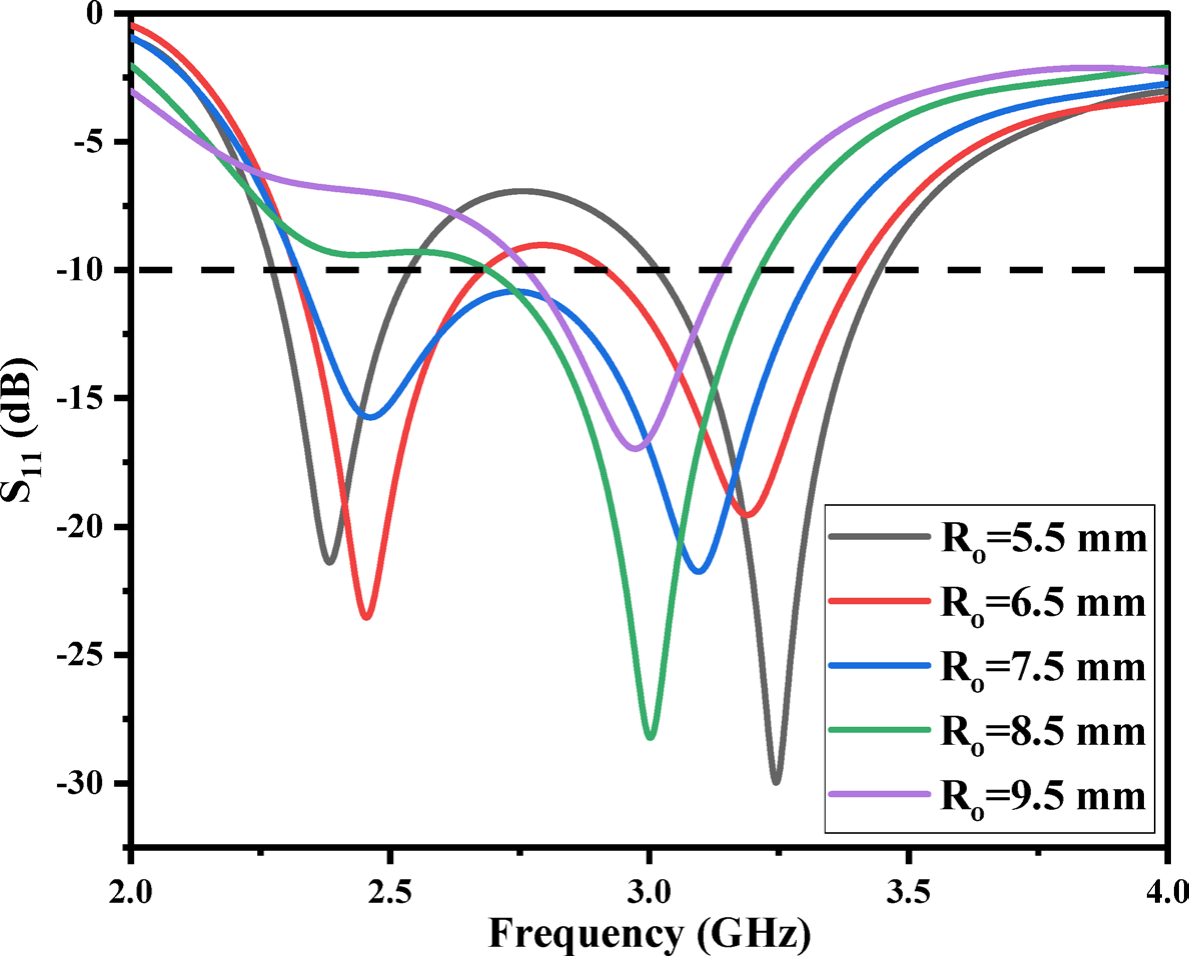
Simulated parametric sweep on the slot radius (R_o_).

Figure 11 shows the effect of varying (R_o_) from 5.5 to 9.5 mm. The outer radius of the ring slot substantially affects both the coupling strength and the frequency position of the upper resonance. As (R_o_) increases, the upper resonance shifts toward lower frequencies, while the relative depths of the lower and upper resonances are redistributed. For smaller or larger values of (R_o_), strong matching may occur at one resonance, but with a less balanced response across the useful frequency range. Therefore, (R_o_ = 7.5) mm was selected because it provides effective coupling to the dielectric resonator and produces a balanced impedance response at both resonant regions. Based on these parametric results, the final values of (L_s_ = 24) mm and (R_o_ = 7.5) mm were adopted in the proposed antenna design.

**Table 3:** Dimensions of the proposed design (mm).

$\:\mathbf{W}$	$\:\mathbf{L}$	h	$\:{\mathbf{D}}_{\mathbf{d}}$	$\:{\mathbf{h}}_{\mathbf{d}}$	$\:{\mathbf{h}}_{\mathbf{m}}$	*R*_o_	$\:{\mathbf{R}}_{\mathbf{i}}$	$\:{\mathbf{W}}_{\mathbf{s}}$	$\:{\mathbf{L}}_{\mathbf{s}}$	$\:{\mathbf{W}}_{\mathbf{f}}$	$\:{\mathbf{L}}_{\mathbf{f}}$
50	50	0.813	30	15	5	7.5	5.5	2.5	24	3.5	30

#### Design analysis

A low-permittivity material is crucial for enhancing the DRA’s bandwidth. It can be used in the multi-segment or stacked DRA system to support dual resonance modes, one associated with the lower permittivity and the other with the higher permittivity. Furthermore, a low-permittivity material can be introduced between the DRA and the feeding circuit to match the DRA impedance to that of the feed.

**Fig. 12:**
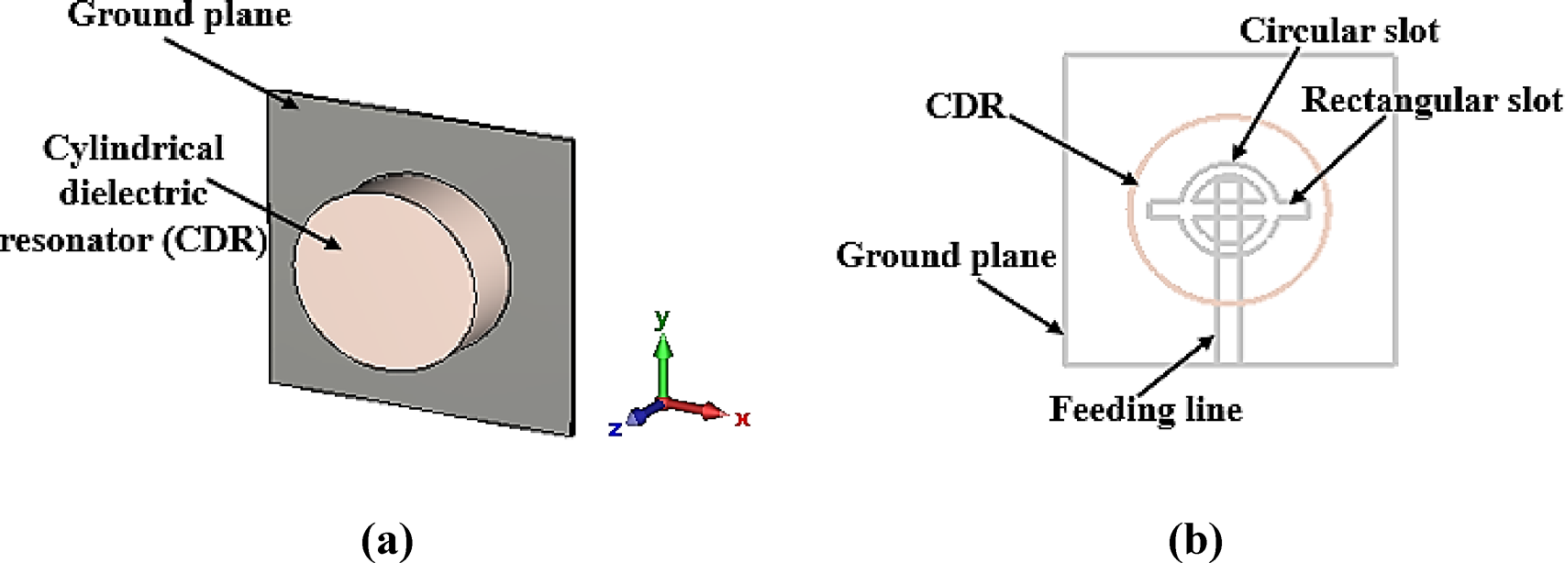
DRA without the matched layer: (**a**) 3D geometry, and (**b**)Transparent 2D geometry.

**Fig. 13:**
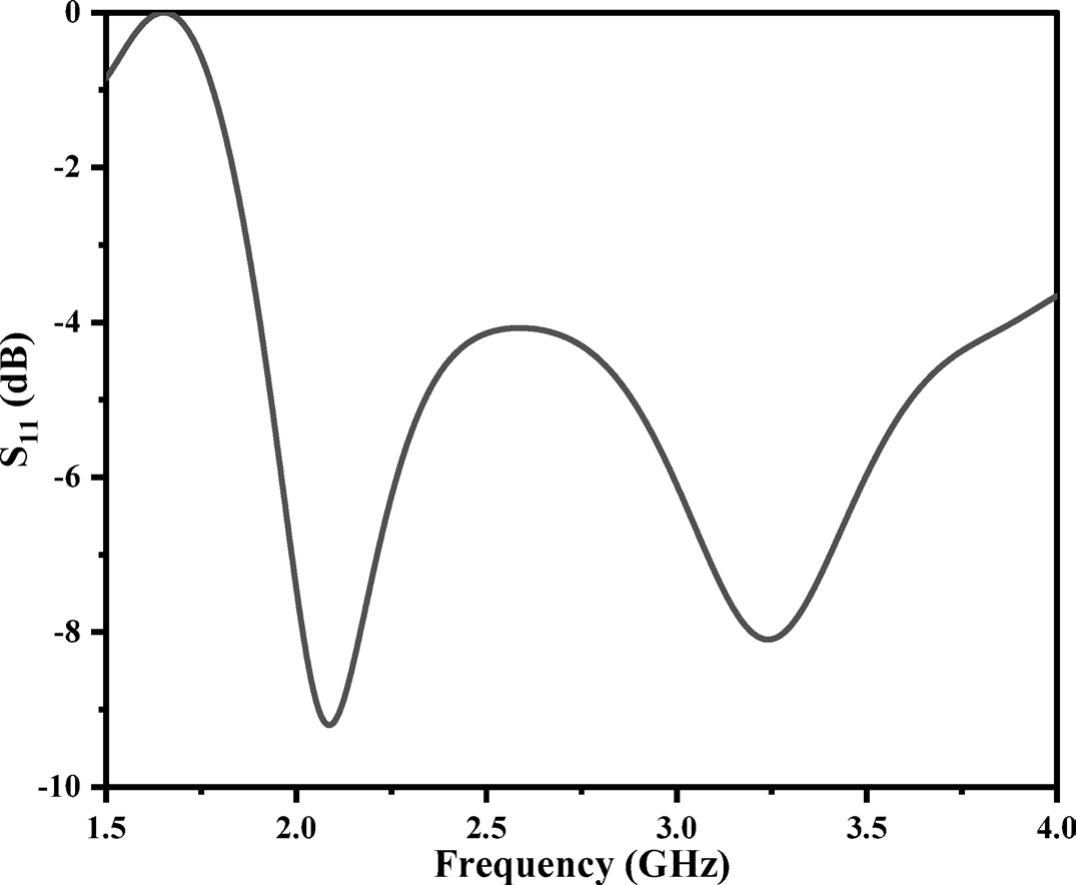
Simulated S_11_ of the DRA without the matched layer.

Firstly, a circular DRA of Rogers 6010 is designed to excite the hybrid EM mode (HEM_11δ_), which resonates at 3.2 GHz. This antenna is fed by a combination of rectangular and ring slots, as depicted in Fig. 12. The proposed antenna was analyzed using full-wave electromagnetic simulations carried out in CST Microwave Studio. The S-parameter results, presented in Fig. 13, demonstrate that the DRA resonates at the HEM_11δ_ mode frequency (3.2 GHz) and at 2.1 GHz, which corresponds to the slots loaded by the DRA. However, as shown in Fig. 13, the matching performance requires further improvement.

**Fig. 14:**
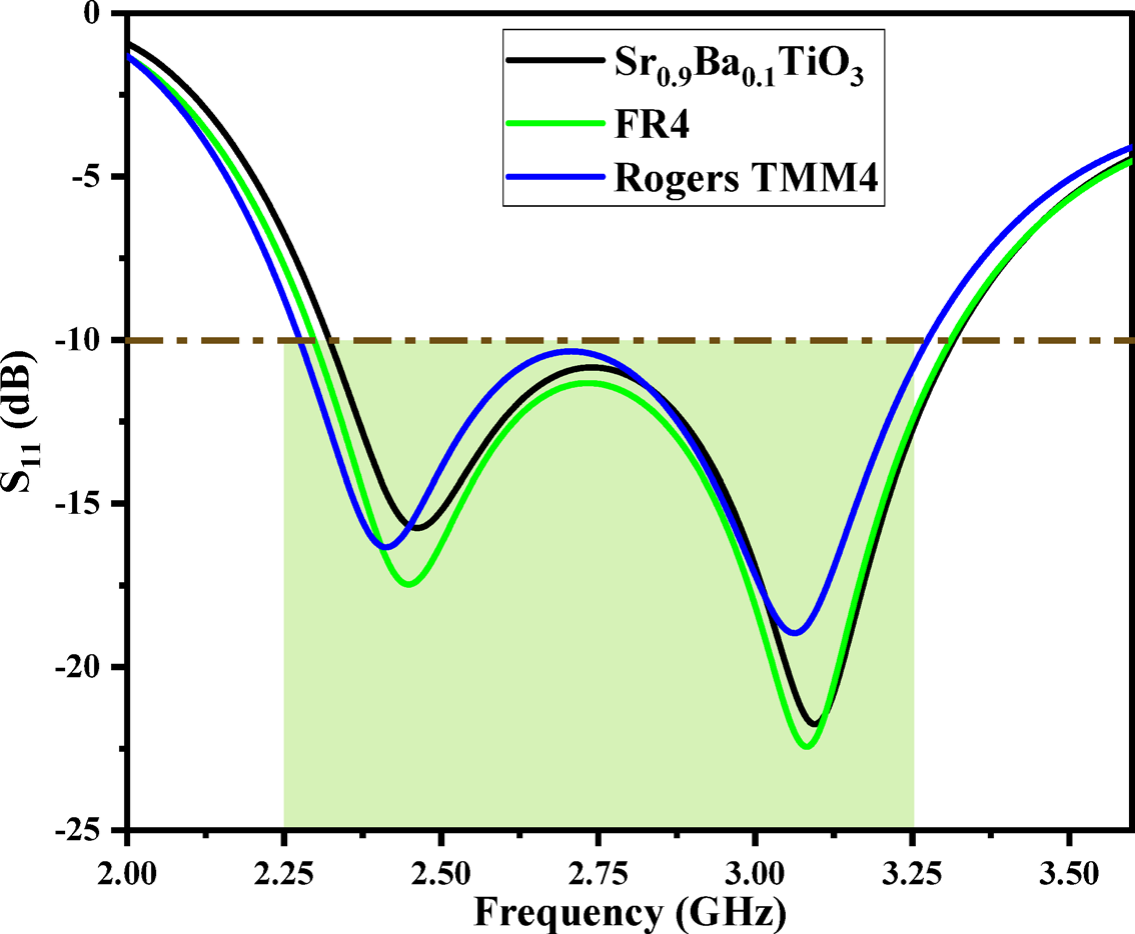
Proposed antenna simulated reflection coefficient for different materials.

**Fig. 15:**
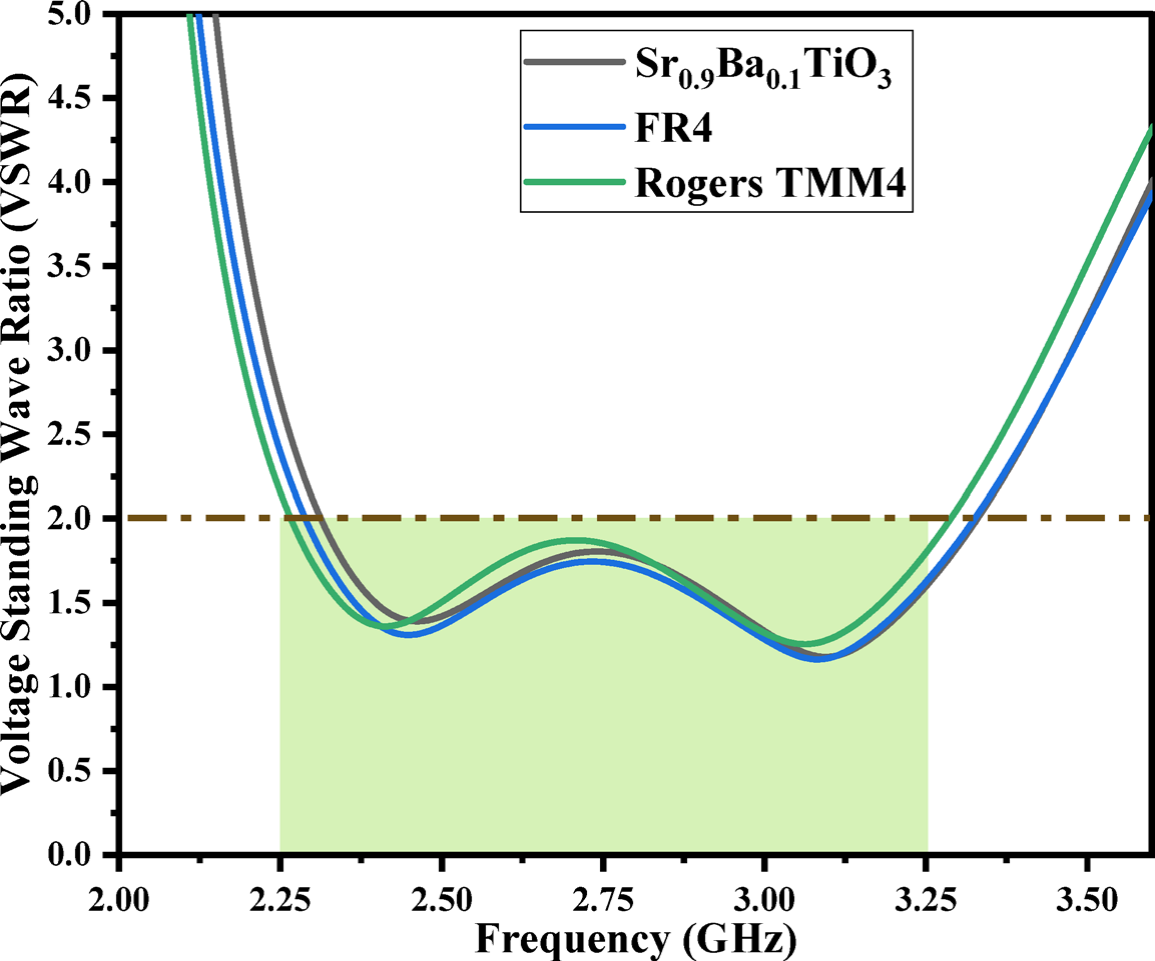
Proposed antenna simulated voltage standing wave ratio for different materials.

**Fig. 16:**
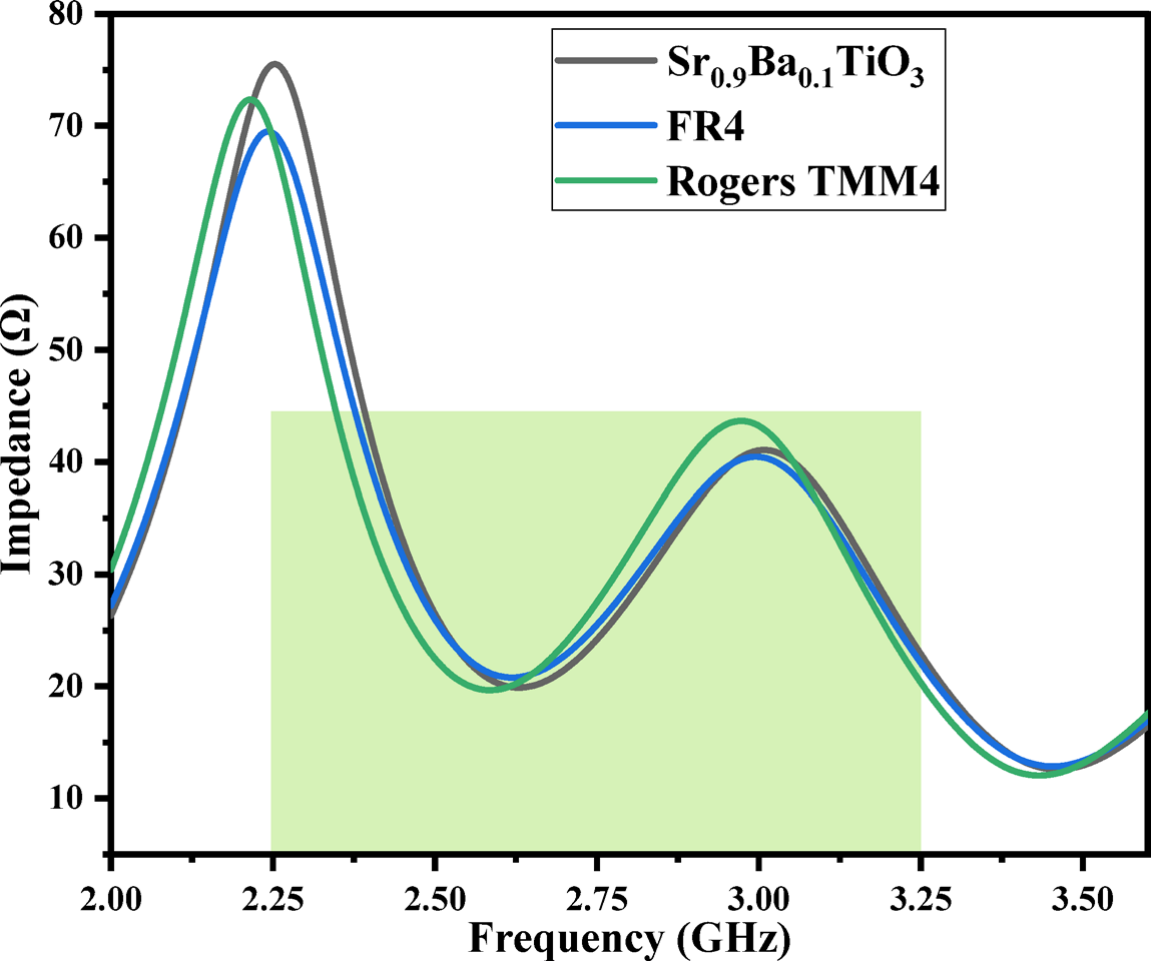
Simulated Z-parameters as a magnitude at the excitation point.

A matching layer is inserted between the DRA and the feeding ground plane to improve impedance matching over a wide frequency range. Three different materials are evaluated as matching layers: (i) the in-house Sr_0.9_Ba_0.1_TiO_3_ disk, whose band-averaged effective ε_r_ ≈ 4.3 and tan δ ≈ 1.6 × 10^−4^ across 2.25–3.25 GHz are read directly from the measured curves of Fig. 8 and imported into the full-wave solver as a frequency-dependent dielectric model; (ii) FR-4 (ε_r_ ≈ 4.3, tan δ ≈ 0.025 at 2 GHz, manufacturer datasheet); and (iii) Rogers TMM4 (ε_r_ = 4.7, tan δ = 0.002 at 10 GHz, manufacturer datasheet). Rogers TMM4 is not used as the main fabricated ceramic under study; rather, it is included as a commercial low-loss microwave reference material to benchmark the matching behavior of Sr_0.9_Ba_0.1_TiO_3_ against a well-established substrate-grade dielectric. As shown in Figs. 14, 15 and 16, the three materials improve the DRA’s response, enabling it to operate effectively within the frequency range of 2.25 GHz to 3.25 GHz.

**Fig. 17:**
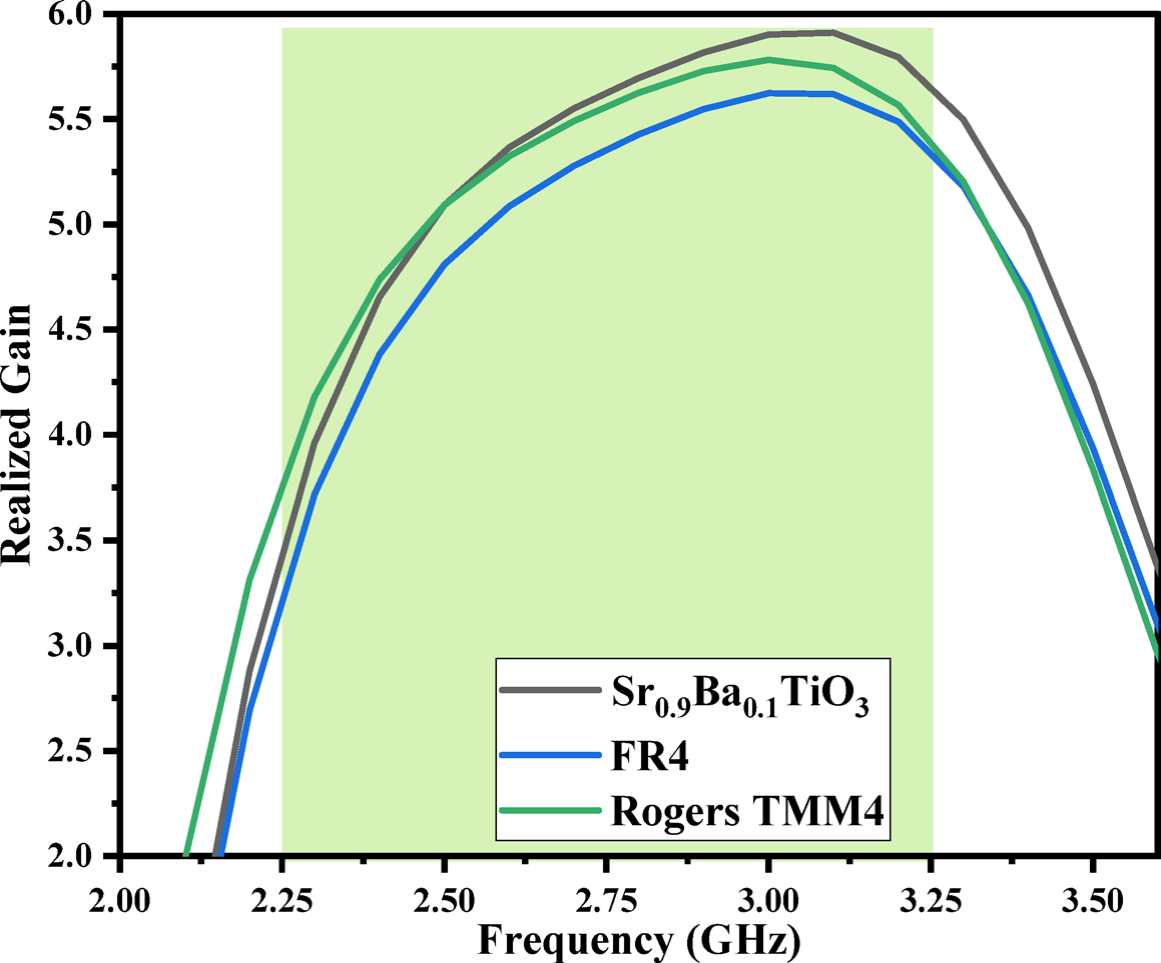
Simulated realized gain of the proposed antenna for different materials.

**Fig. 18:**
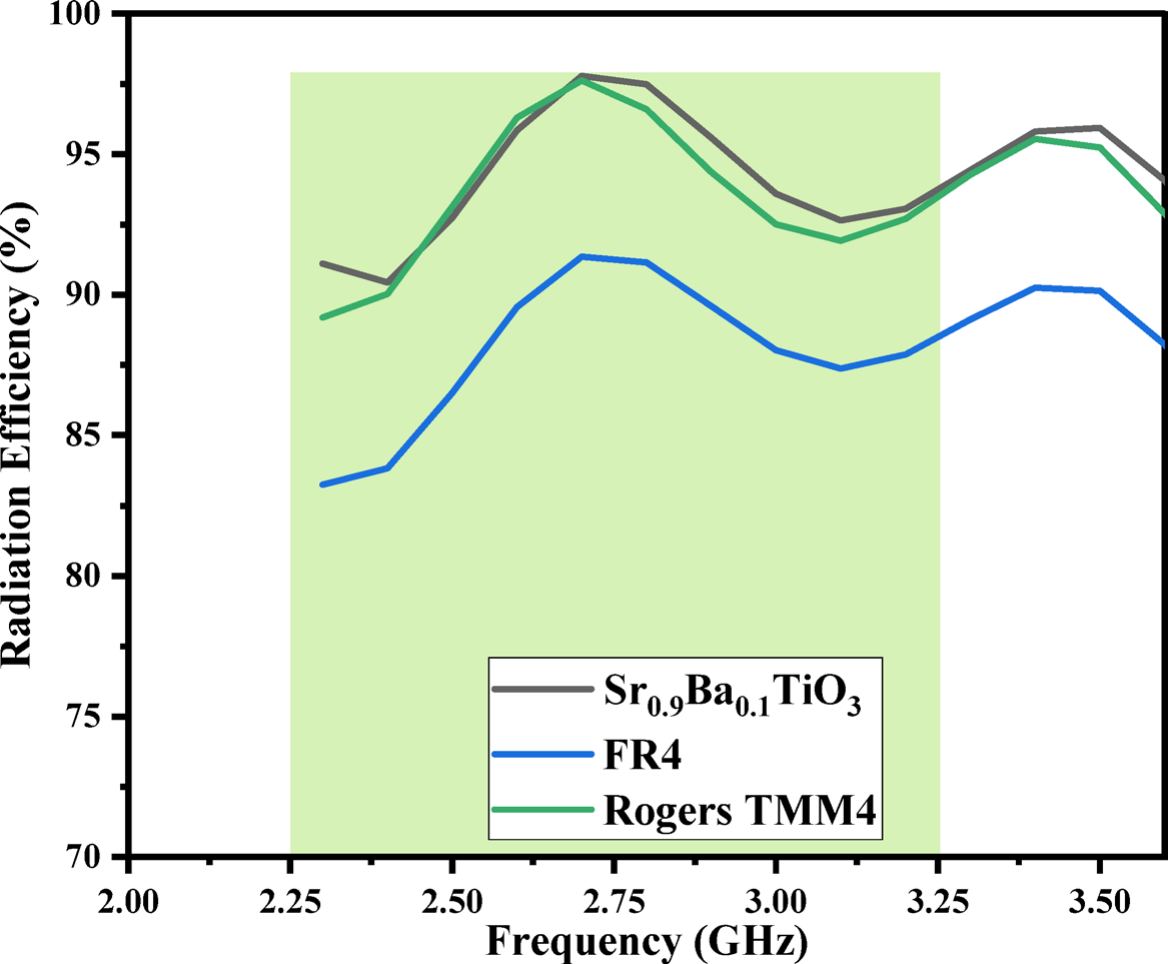
Simulated radiation efficiency of the proposed antenna for different materials.

**Fig. 19:**
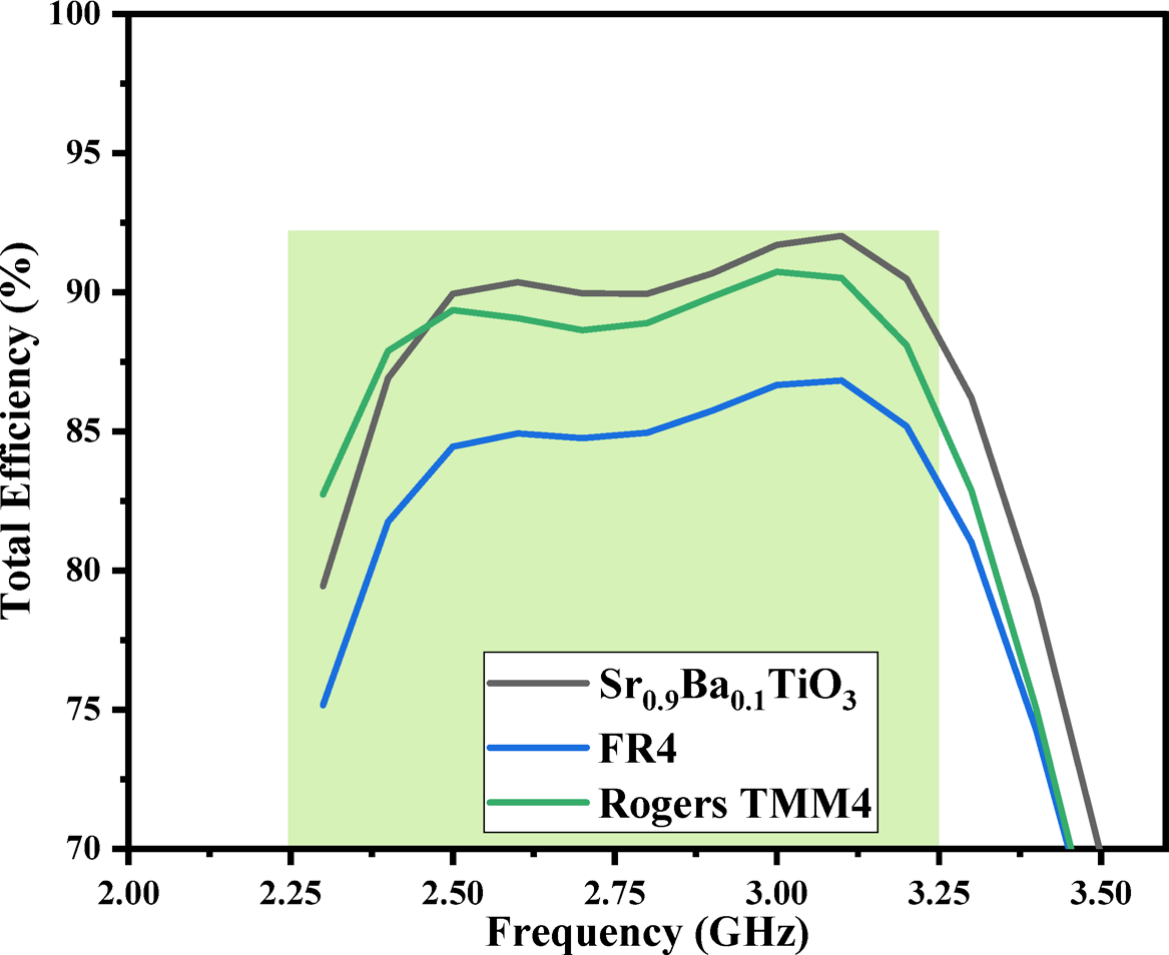
Simulated total efficiency of the proposed antenna for differentmaterials.

**Fig. 20:**
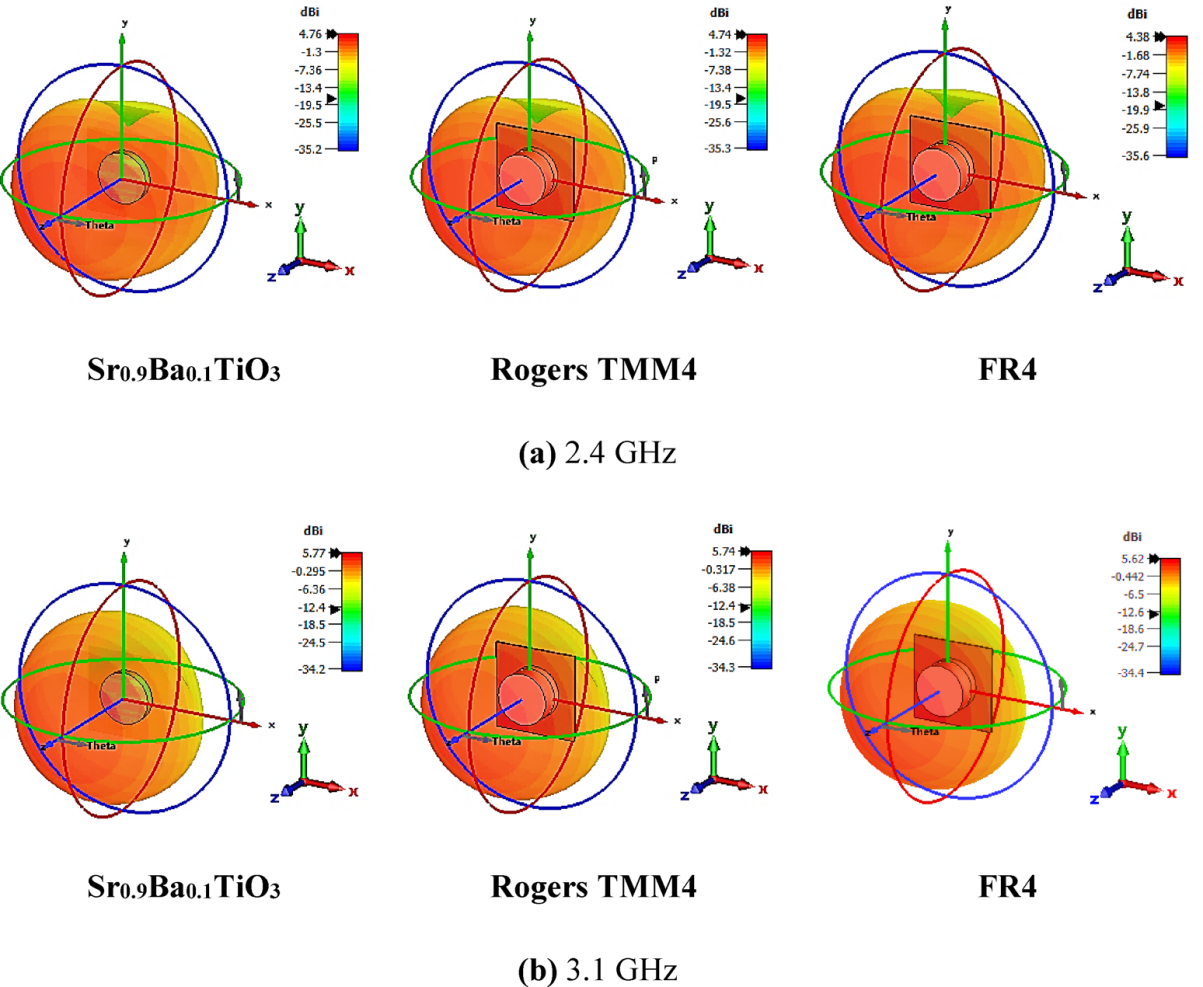
Simulated 3D radiation patterns of the DRA with a lower-permittivity layer for different materials.

**Fig. 21:**
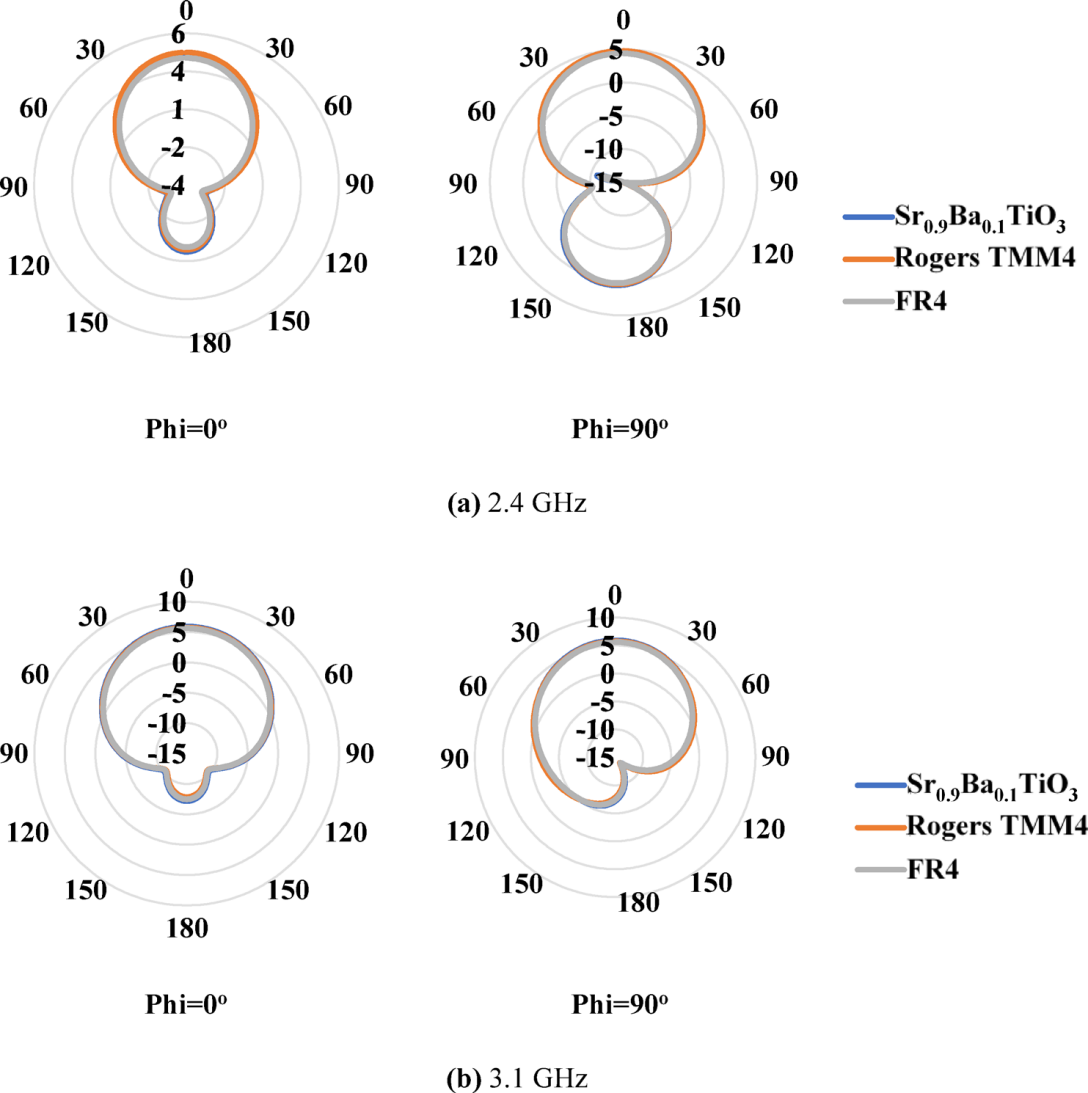
Simulated 2D radiation patterns of the DRA with a lower-permittivity layer for different materials.

Although FR4 and Rogers TMM4 exhibit similar impedance-matching behavior, Rogers TMM4 offers better gain and efficiency due to its lower loss tangent, as shown in Figs. 17, 18, and 19. Compared with both materials, Sr_0.9_Ba_0.1_TiO_3_ outperforms them by achieving superior impedance matching, gain, and efficiency while maintaining operation over the same bandwidth, as illustrated in Figs. 14, 15, 16, 17, 18, and 19. Additionally, the 3D radiation patterns in Fig. 20 further confirm that Sr_0.9_Ba_0.1_TiO_3_ delivers the best overall performance, making it the most effective and cost-efficient choice for the matching layer between the DRA and the ground plane. To facilitate comparison of the radiation characteristics of the proposed antenna, the 2D radiation patterns in the principal planes (E-plane and H-plane) are illustrated in Fig. 21 at representative resonant frequencies. The obtained results confirm that the proposed DRA exhibits stable radiation behavior with acceptable directional characteristics across the operating band.

**Fig. 22:**
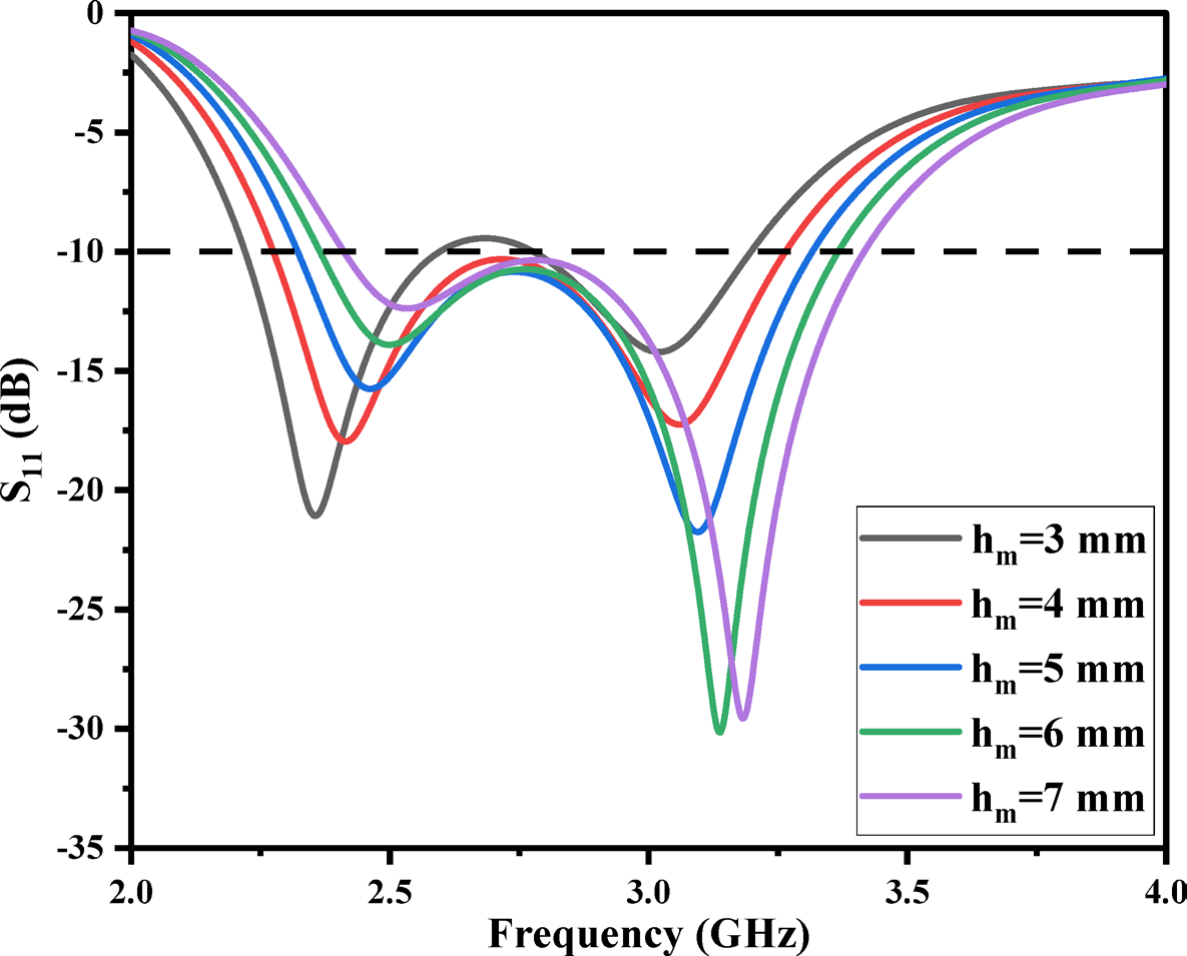
Simulated parametric sweep of S_11_ for the matching layer thickness (h_m_).

**Fig. 23:**
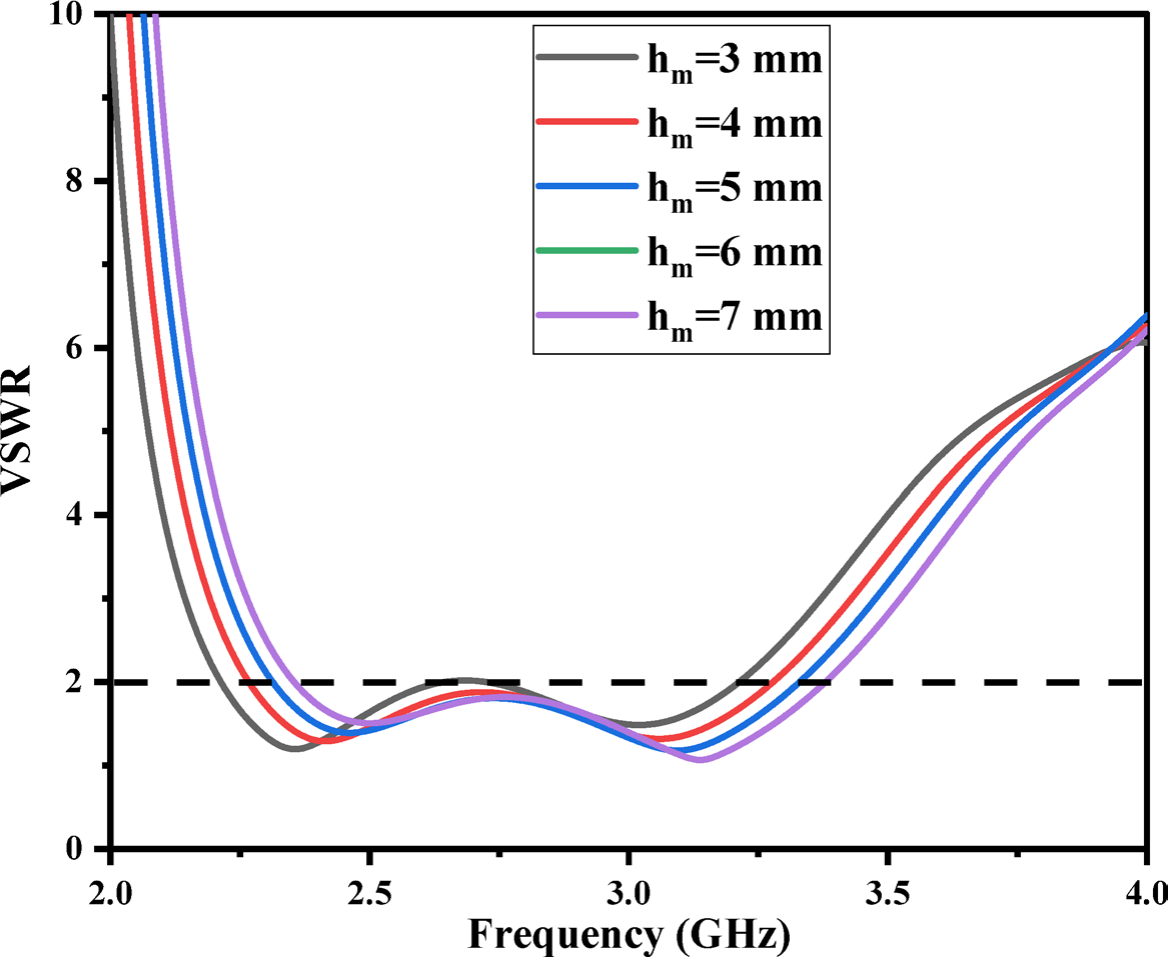
Simulated parametric sweep of VSWR for the matching layer thickness (h_m_).

**Fig. 24:**
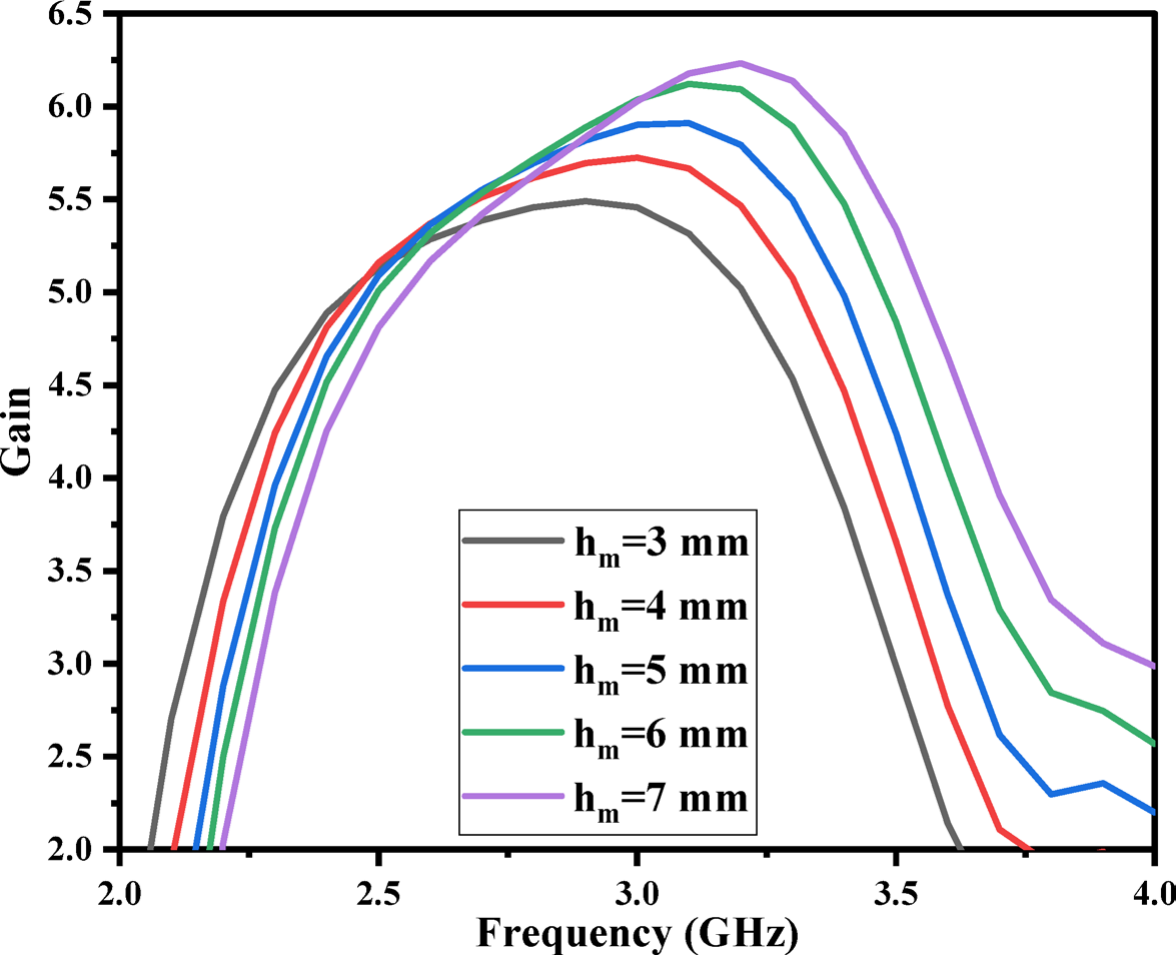
Simulated parametric sweep of realized gain for the matching layer thickness (h_m_).

To investigate the effect of the matching-layer thickness on DRA performance, a parametric study was conducted by varying hm from 3 to 7 mm while maintaining the remaining geometrical and material parameters unchanged. The simulated S_11_, VSWR, and gain characteristics are presented in Figs. 22, 23 and 24, respectively. It is observed that changing h_m_ alters the electromagnetic coupling between the dielectric resonator and the feeding structure, leading to variations in both resonance depth and radiation gain. Increasing h_m_ generally improves the matching around the upper resonance near 3.1–3.2 GHz and increases the maximum gain. In particular, thicker layers of 6 and 7 mm provide deeper S_11_ minima and slightly higher gain values; however, this improvement is accompanied by an increased antenna profile and a shift in the resonance characteristics. Accordingly, h_m_ = 5 mm was selected as a suitable design compromise, as it maintains a compact structure while providing satisfactory impedance matching, with S_11_ below − 10 dB and VSWR below 2 within the useful operating regions, and a peak gain of approximately 5.9 dBi.

**Fig. 25:**
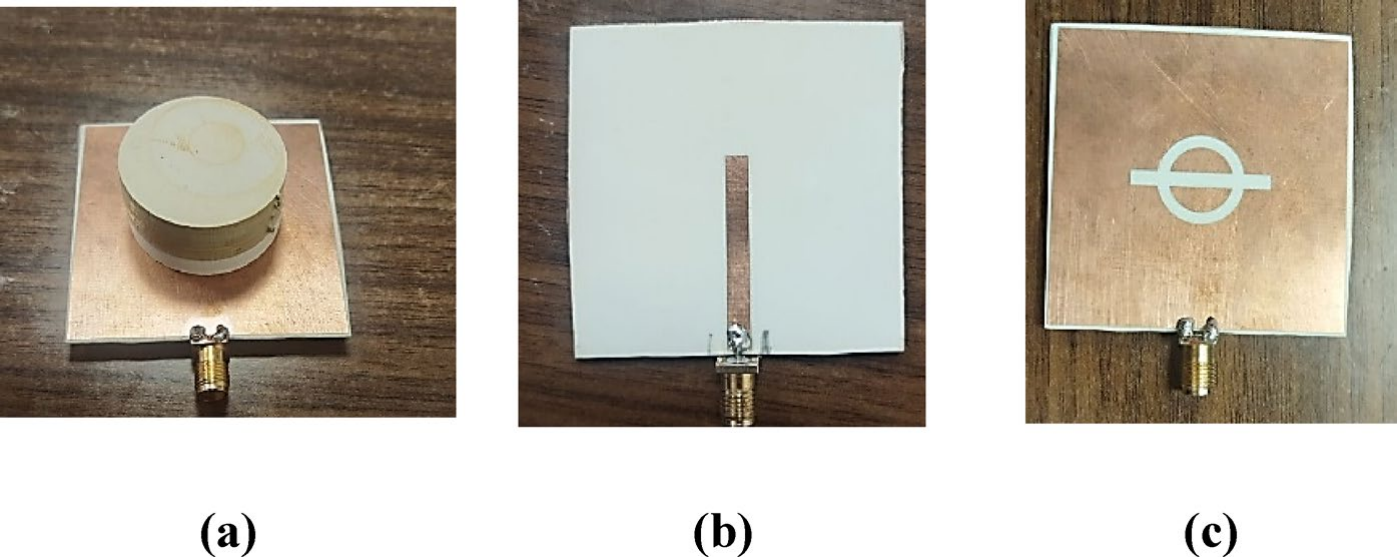
Fabricated prototype: (**a**) Full structure, (**b**) Feeding microstrip line, and (**c**) Excitation slot.

**Fig. 26:**
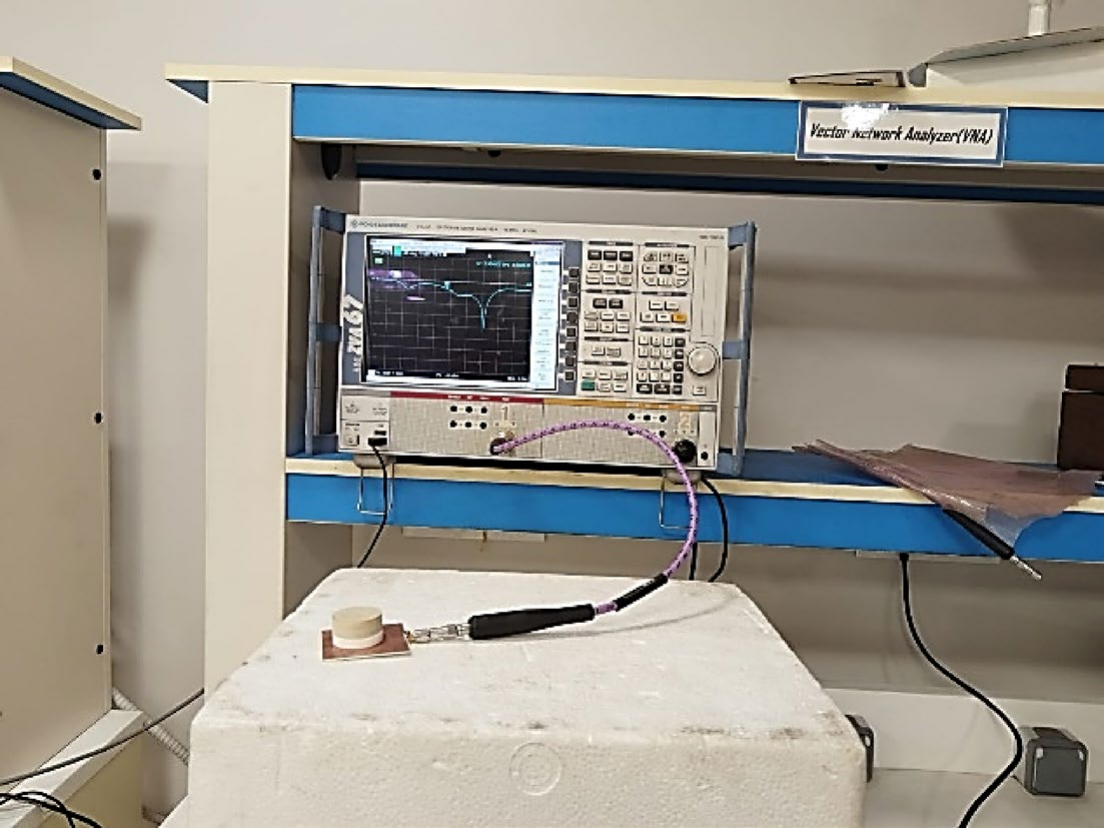
Measurement setup.

**Fig. 27:**
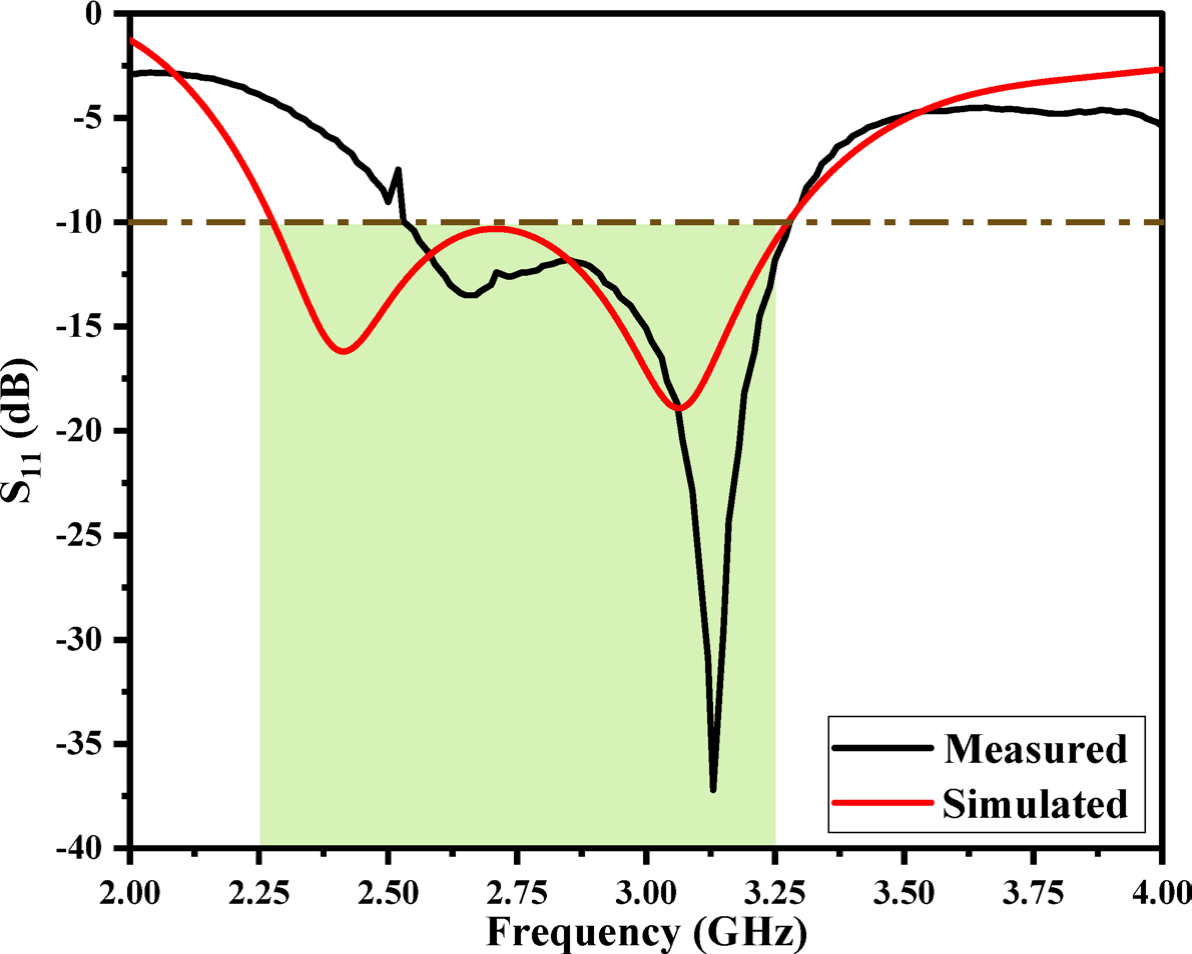
Simulation and measurements of the proposed antenna’s reflection coefficient (S_11_).

Figure 25 shows the manufactured layer structure of the resonator with the Rogers 4003 substrate, the Sr_0.9_Ba_0.1_TiO_3_ sample substrate, and the Rogers 6010 top layer. During prototype assembly, the Rogers 4003 substrate carrying the microstrip feed and defected ground structure was used as the base layer. The Sr_0.9_Ba_0.1_TiO_3_ ceramic layer was carefully positioned above the ground plane at the designed location, followed by the Rogers 6010 cylindrical dielectric resonator. The layers were aligned coaxially according to the simulated geometry to preserve the intended electromagnetic coupling. In the present prototype, the layers were mechanically fixed during measurement to maintain stable contact and alignment. No conductive adhesive was introduced between the dielectric layers to avoid additional parasitic loss or unintended perturbation of the electromagnetic field distribution. However, small interfacial air gaps or surface roughness between the stacked dielectric materials may still exist and can contribute to minor differences between the simulated and measured reflection responses. The slot and the microstrip line configurations are presented in Fig. 25b and c, which describe the device’s geometric features. Figure 26 shows the measurement setup for the reflection coefficient using the Rohde & Schwarz ZNA 76 vector network analyzer. The measured and simulated reflection coefficients are plotted in Fig. 27. The data indicate that the resonator operates at maximum power at certain frequencies. A slight discrepancy is observed between the simulated and measured reflection responses in the 2–2.75 GHz range. This difference is expected because the numerical model represents an idealized antenna structure with nominal dimensions, uniform material properties, and ideal electrical contact between the DRA, the matching layer, the substrate, and the ground plane. In the fabricated prototype, several practical factors may affect the impedance response, including small dimensional deviations during fabrication, surface roughness of the resonator, possible air gaps at the dielectric interfaces, uncertainty in the frequency-dependent dielectric constant and loss tangent of the Sr_0.9_Ba_0.1_TiO_3_ sample, and parasitic effects introduced by the SMA connector and soldering region. These effects alter the effective dielectric loading and input impedance, resulting in a resonance shift and amplitude variations in the measured response. Nevertheless, the simulated and measured results show the same overall operating trend, confirming the validity of the proposed DRA configuration.

The sensitivity of the simulated antenna response to dielectric-property uncertainty was tested by re-running the full-wave simulation at the four corners of an (ε_r_ ± 3%, tan δ ± 10%) envelope around the measured values. The resulting |S_11_|(f) envelope brackets the measured curve across most of the 2.25–3.25 GHz band, but does not bracket the residual band-localized mismatch over 2.0–2.75 GHz. The latter discrepancy, therefore, cannot be attributed solely to material-property uncertainty and is consistent with the fabrication-tolerance sources enumerated above.

**Fig. 28:**
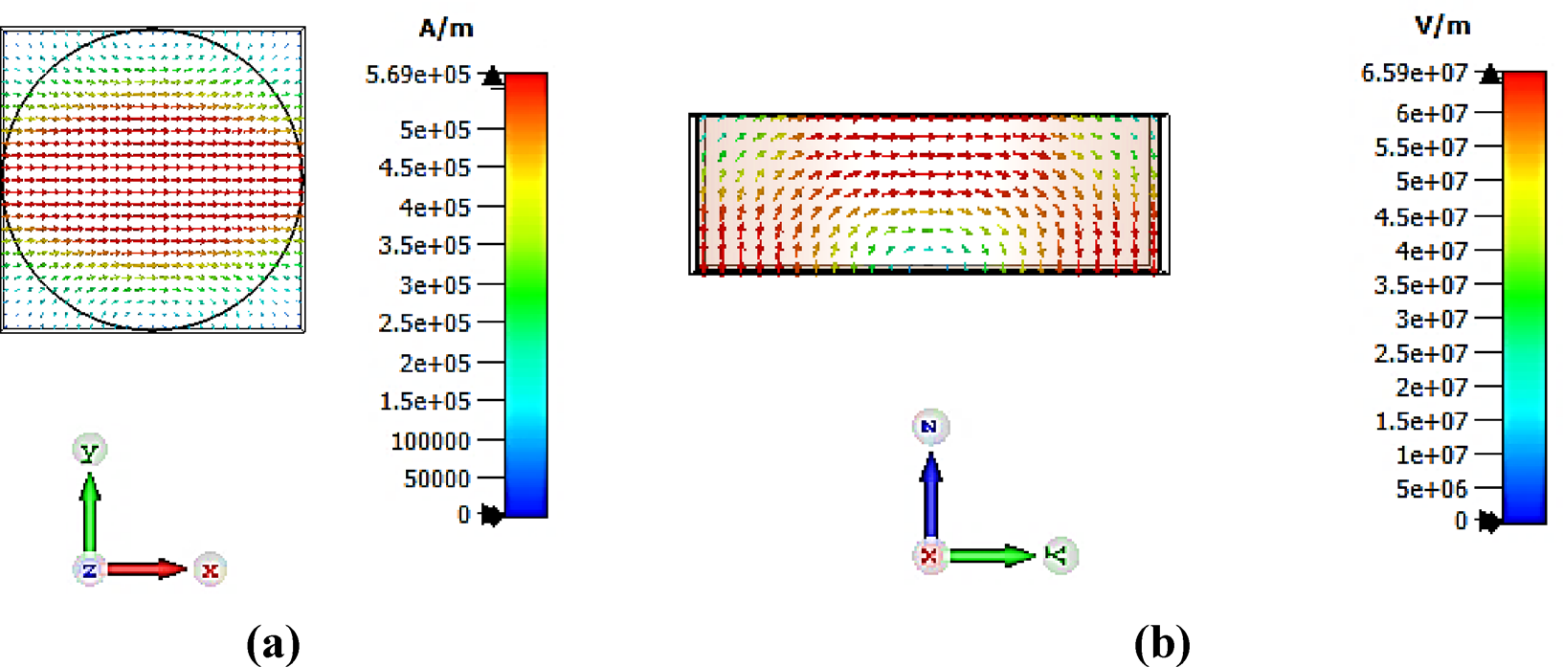
Simulated HEM_11δ_ field distributions at 3.2 GHz: (**a**) Magnetic Field, and (**b**) Electric Field.

The field patterns shown in Fig. 28 reveal the EM characteristics within the resonator, demonstrating strong field confinement and effective energy coupling. The comparison between the experimental and simulation results indicates tolerable agreement. The minute differences between the simulation and measured outcomes can be attributed to solder joints and fabrication tolerances. Manufacturing imperfections can cause minute variations, leading to mismatches between simulated and real outputs. The hybrid of Rogers 4003 and Rogers 6010 with the Sr_0.9_Ba_0.1_TiO_3_ sample provides a balanced dielectric constant and loss tangent, which render the resonator’s superior performance. The rectangular and circular slots improve the EM characteristics of the resonator by minimizing undesirable modes and enhancing signal integrity.

The present design occupies a distinct position within the recent ceramic-based DRA literature. References^2,6,10,12^ employ a single ceramic, Ba_0.7_Sr_0.3_TiO_3_, a Mn-substituted garnet, Ce(Nb, V)NbO_4_, and a BaTiO_3_/V_2_O_5_ composite, respectively, as the resonator core, and operate at sub-6 GHz, mm-wave, C-band, and X-band center frequencies. The present antenna, in contrast, uses the in-house Sr_0.9_Ba_0.1_TiO_3_ ceramic as a matching layer between commercial laminates and operates in the 2.25–3.25 GHz sub-6 GHz 5G band. Within the sub-6 GHz subset most directly comparable to the present design, the achieved matched bandwidth (1.0 GHz, fractional bandwidth ≈ 36%) and the measured radiation performance are consistent with the values reported for single-ceramic-resonator approaches operating in the same band. In contrast, the ceramic ε_r_ required by the matching-layer design philosophy adopted here (effective ε_r_ ≈ 4.3) is roughly an order of magnitude lower than that required for ceramic-resonator-core designs (ε_r_ ~ 45–50 in refs^8,12^).

## Conclusions

This study illustrates the strategic synthesis and characterization of Sr_1−x_Ba_x_TiO_3_ (0.0 ≤ x ≤ 0.4) nanocrystals for microwave dielectric applications in dielectric resonator antennas, critical to future 5G/6G technologies. Extensive structural and dielectric analysis concluded that Sr_0.9_Ba_0.1_TiO_3_ is the best, with reasonable permittivity, low dielectric loss, and exemplary temperature stability. Including an optimally optimized multi-layered cylindrical DRA structure with Rogers 4003 and 6010 substrates improved antenna performance, with exemplary impedance matching, wide gain, and high radiation efficiency within the 2–5 GHz frequency band. Within the prototype stack, Sr_0.9_Ba_0.1_TiO_3_ is used specifically as the matching layer between the slot-fed Rogers 4003 ground laminate and the Rogers 6010 cylindrical dielectric resonator core. The high-ε_r_ resonator function is filled by Rogers 6010; SBTO is not used as the resonator body itself. The composition-engineered SBTO data therefore inform the choice and design of the matching layer, while the resonator and feed/ground geometry are realized in commercially available laminates. Experimental verification ensured successful simulation-experiment corroboration, validating the design method’s validity and performance. These results demonstrate the untapped potential of Ba-substituted SrTiO3 ceramics for microwave communication devices and lay the roadmap for future miniature wireless antenna devices with higher performance.

## Supplementary Information

Below is the link to the electronic supplementary material.

Supplementary Material 1

## Data Availability

The datasets used and/or analysed during the current study are available from the corresponding author on reasonable request.
