# Supplementary material for: Composition-engineered Sr1−xBaxTiO3 for high-efficiency dielectric resonator antennas in 5G/6G bands
Source: Sci Rep. 2026 Jul 14;16:22034. doi: 10.1038/s41598-026-61044-1 (PMC13369934; doi:10.1038/s41598-026-61044-1)
Supplement: Supplementary file 1 — Supplementary Material 1 [file 41598_2026_61044_MOESM1_ESM.docx]

**Supplementary File**

**Composition-Engineered Sr_1-x_Ba_x_TiO_3_ for High-Efficiency Dielectric Resonator Antennas in 5G/6G Bands**

Moustafa A. Darwish^a*^, F. Fakhry^a^ , Marwa M. Hussein^a^, Yousef M. Abd El-Maboud^a^, Enas H. El-Ghazzawy^a^, Anwer S. Abd El-Hameed^b^, Asmaa I. Afifi^b^, Sherif G. Elsharkawy^c^, M. M. Salem^a^

^a^ Physics Department, Faculty of Science, Tanta University, Tanta 31527, Egypt

^b^ Electronics Research Institute, Giza 12622, Egypt

^c^ Basic and Applied Sciences, College of Engineering and Technology, AASTMT, Alexandria 1029, Egypt.

**XRD**

The X-ray diffraction (XRD) patterns and corresponding refinements for the synthesized ferroelectric nanoparticles Sr_1-x_Ba_x_TiO_3_ with compositions x = 0.0, 0.1, 0.2, 0.3, and 0.4 are shown in Fig. S1. These patterns demonstrate the formation of a single-phase cubic perovskite structure. Specifically, for the undoped sample SrTiO_3_ (x = 0.0), distinct diffraction peaks were observed at 2θ angles corresponding to crystallographic planes (hkl) at 22.76° (100), 32.37° (110), 39.92° (111), 46.45° (200), 52.18° (210), 57.74° (211), 67.75° (220), and 77.07° (310). These characteristic peaks confirm the formation of the cubic perovskite phase of SrTiO_3_, matching closely with standard reference data (JCPDS card numbers 35–0734 and 79–0176) and are attributed to the Pm-3m (221) space group [27,28]. Similarly, the XRD patterns for Ba-doped samples are displayed in Fig. S1. The observed diffraction peaks consistently correspond to the aforementioned crystallographic planes, reinforcing the presence of a cubic crystal structure. These peaks are in good agreement with standard data for Sr_0.8_Ba_0.2_TiO_3_ (JCPDS card number 01-080-4496) and Sr_0.6_Ba_0.4_TiO_3_ (JCPDS card number 01-080-4495), both of which are categorized within the space group Pm-3m (No. 221) [28].

While the sharp, narrow diffraction peaks indicate the high-quality crystalline nature of the synthesized nanoparticles, the slight peak broadening observed upon substituting barium can be attributed to induced lattice stress, a phenomenon that will be elaborated upon subsequently. As the barium content increases, a modest shift to lower 2θ in the diffraction angles (2θ) is also noticeable in Fig. S1. This shift arises from alterations in the d-spacing, which are directly associated with variations in the lattice parameters resulting from incremental barium incorporation [28].

The refinement of the diffraction patterns was performed using the Rietveld method facilitated by Fullprof software. Atomic positions were held fixed throughout the refinement, and the background was modeled by linear interpolation between selected background points and refined peaks. Peaks were fitted utilizing the Thomson-Cox-Hastings pseudo-Voigt analytical function. Initial refinement cycles focused primarily on optimizing scale factors and background parameters. Subsequently, lattice constants, scale factors, peak shape parameters, temperature factors, and atomic site occupancies were treated as free parameters and systematically refined [29].

R_exp_ and R_wp_ are the expected and weighted profile R-factors, respectively, used to assess the quality of Rietveld fits. These values should be less than 10%. In addition, the goodness-of-fit (GoF = R_wp_/R_exp_) and the χ^2^ statistic should tend to be 1 [30]. It is important to note that the best fit to the experimental diffraction data is achieved when these parameters have low values, as shown in Table S1.

**Table S1:** The expected (R_exp_) and weighted (R_wp_) profile R-factors, the GoF, and χ^2^ from the Rietveld analysis of Sr_1-x_Ba_x_TiO_3_ (0.0 ≤ x ≤ 0.4) samples.

| Sample | R_exp_ | R_wp_ | GoF | χ^2^ |
| --- | --- | --- | --- | --- |
| SrTiO_3_ | 7.04 | 14.8 | 2 | 4.41 |
| Sr_0.9_Ba_0.1_TiO_3_ | 6.77 | 16.8 | 2.48 | 6.13 |
| Sr_0.8_Ba_0.2_TiO_3_ | 7.47 | 20.1 | 2.69 | 7.2 |
| Sr_0.7_Ba_0.3_TiO_3_ | 7.4 | 18.9 | 2.55 | 6.53 |
| Sr_0.6_Ba_0.4_TiO_3_ | 6.97 | 15 | 2.15 | 4.64 |

|  |  |
| --- | --- |
|  |  |
|  | |
| **Fig. S1**. XRD patterns and Rietveld refinements of Sr_1-x_Ba_x_TiO_3_ (0.0 ≤ x ≤ 0.4) samples. | |

The crystallite size (*β_d_*) and the lattice strain (*β_ε_*) cause line broadening (*β_hkl_*), and consequently *β_hkl_* = *β_d_* + *β_ε_*. *β_d_* represents the crystallite-size broadening, while *β_ε_* represents the strain-induced broadening arising from local distortions of the crystalline lattice relative to the ideal crystalline structure. The mean crystallite size, R, is determined from the Debye–Scherrer equation: *β_d_* = 0.94λ/Rcosθ (=1.54 Å), and the strain, ε, is determined from *β_ε_* = 4εtanθ based on the Williamson-Hall equation [29]:

$$\beta_{hkl}\cos\theta=\beta_{d}+\beta_{\varepsilon}=\frac{0.94\lambda}{R}+4\varepsilon sin\theta\to(1)$$

β_hkl_ is the full width at half maximum (FWHM) of the instrumentally corrected broadening, obtained from the Rietveld refinement of the FWHM parameters. By plotting β_hkl_ cosθ as a function of$sin\theta$ (see Fig. S2), R and ε were calculated from the intersection of the straight line with the Y-axis and the slope, respectively.

|  |
| --- |
| **Fig. S2** Williamson-Hall plot of Sr_1-x_Ba_x_TiO_3_ (0.0 ≤ x ≤ 0.4) samples. |

Furthermore, it is crucial to note that the crystallite size estimated by the Williamson-Hall method differs from that obtained using Scherrer's formula. This discrepancy arises because the Williamson-Hall analysis accounts for strain-induced broadening, whereas Scherrer's formula does not include this strain correction factor. As the Ba substitution ratio (x) increases, the crystallite size (R) generally decreases from 133 nm to 47 nm, leading to enhanced lattice distortions at grain boundaries and within crystallites, consequently elevating lattice micro-strain (ɛ), as detailed in Table S2.

The refined lattice constant a shows a small initial contraction at x = 0.1 (3.9126 → 3.9017 Å), followed by a monotonic expansion for x ≥ 0.2 (3.9017 → 3.9400 Å at x = 0.4), consistent with the gradual A-site accommodation of the larger Ba^2+^ ion (r_XII_ = 1.61 Å) replacing Sr^2+^ (r_XII_ = 1.44 Å). The slight initial dip at x = 0.1 reflects the competing influence of lattice strain induced by dislocations and grain boundaries, which is more pronounced at low Ba content, where the heavier Ba^2+^ is dilute, before the larger-ion expansion effect dominates at higher x.

In BaTiO_3_ amorphous samples, nucleation-controlled crystallization occurred, whereas in SrTiO_3_, the microstructure after massive, globular crystallites indicated a non-nucleation-controlled crystallization. As the Sr concentration increases in the solid solution Sr_1-x_Ba_x_TiO_3_, the fine equiaxed crystallites in BaTiO_3_ will transform into coarse globular crystallites in SrTiO_3_. Moreover, nucleation and grain-size expansion occurred after the SrTiO_3_ sample crystallized. Since the ionic radius of Sr^2+^ is smaller than that of Ba^2+^, as Sr content increases, improved diffusion may lead to greater grain development [31].

The X-ray density (D_x_) was calculated as well using the formula below [32,33]:

$$D_{x}=\frac{\mathrm{MZ}}{N_{A}a^{3}} \to(2)$$

Where (M) represents the molecular weight of the sample, (Z) = 1 for the cubic structure, and (N_A_) represents Avogadro's number. D_x_ increases when the molecular weight of the samples increases. The increase in molecular weight resulting from incorporating Ba typically indicates the incorporation of heavier atoms into the material. Hence, this increased the atomic density, thereby contributing to a higher D_x_. The calculated density (D) of the compressed discs of the ferroelectric samples has also been calculated by [32,33]:

$$D=\frac{m}{Vol.} \to(3)$$

Where (m) represents the sample mass and (Vol.) the volume of the disc sample. Additionally, porosity (P) has been determined by [32,33]:

$$P=1-\frac{D}{D_{x}} \to(4)$$

The dislocation density, denoted as δ and defined by the number of dislocation lines per volume of the crystal, can be calculated by the equation given below [32,33]:

$$\delta=\frac{1}{R^{2}} \to(5)$$

The dislocation density increased with increasing lattice strain. (ɛ) and (δ) increase with a reduction in crystallite size. This occurs because small crystals have larger grain boundaries, which slow down the movement of dislocations. Thus, these dislocations become confined to the grain boundaries of the smaller crystallites. Peak broadening increases in smaller crystallites due to the reduced coherence length imposed by dislocations. All the calculated values mentioned above are listed in Table S2.

**Table S2:** Structural and microstructural parameters of Sr_1-x_Ba_x_TiO_3_ (0.0 ≤ x ≤ 0.4) samples.

| Sample | a  (Å) | R (nm)  Average particle size from XRD | D  (kg/m^3^) | D_x_  (kg/m^3^) | P (%) | δ  (m^-2^) | ε  (lin^−2^ m^−4^) |
| --- | --- | --- | --- | --- | --- | --- | --- |
| SrTiO_3_ | a=3.9126 | 133.0518 | 2978.37 | 5087.2 | 41.4 | 5.65 ×10^13^ | 0.0035 |
| Sr_0.9_Ba_0.1_TiO_3_ | a=3.9017 | 162.6189 | 2926.90 | 5268.6 | 44.4 | 3.78× 10^13^ | 0.0020 |
| Sr_0.8_Ba_0.2_TiO_3_ | a=3.9155 | 58.5428 | 3117.10 | 5350.1 | 41.7 | 2.92× 10^14^ | 0.0041 |
| Sr_0.7_Ba_0.3_TiO_3_ | a=3.9244 | 91.4731 | 2821.84 | 5450.2 | 48.2 | 1.19× 10^14^ | 0.0047 |
| Sr_0.6_Ba_0.4_TiO_3_ | a=3.9400 | 47.2119 | 3133.15 | 5520.7 | 43.2 | 4.49× 10^14^ | 0.0053 |

The cubic crystal structure for the Pm-3m space group was determined using the VESTA software program, with CIFs obtained from Rietveld analyses of each sample, as shown in Fig. S3 and Fig. S4. The bond lengths of Ba/Sr–O and Ti–O were calculated and found to generally increase with increasing Ba content, with some non-monotonic variation attributable to local strain effects, as presented in Table S3. The bond length affects the vibrational frequency, as discussed later.

**Table S3:** Sr–O and Ti–O bond lengths (Å) in Sr_1-x_Ba_x_TiO_3_ (0.0 ≤ x ≤ 0.4) samples.

| Sample | Sr/Ba–O | Ti–O |
| --- | --- | --- |
| SrTiO_3_ | 2.76659 | 1.95627 |
| Sr_0.9_Ba_0.1_TiO_3_ | 2.77438 | 1.96251 |
| Sr_0.8_Ba_0.2_TiO_3_ | 2.76870 | 1.95777 |
| Sr_0.7_Ba_0.3_TiO_3_ | 2.77498 | 1.96221 |
| Sr_0.6_Ba_0.4_TiO_3_ | 2.78597 | 1.96997 |

| 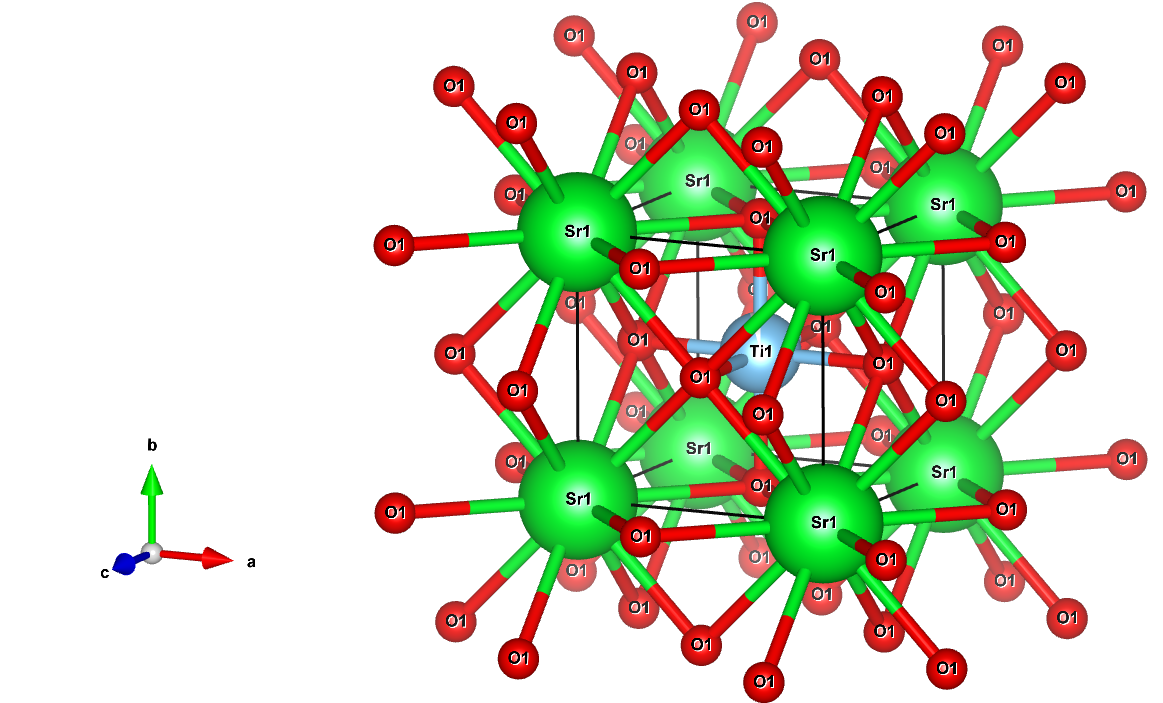 |
| --- |
| **Fig. S3** Cubic crystal structure of SrTiO_3_. |
| 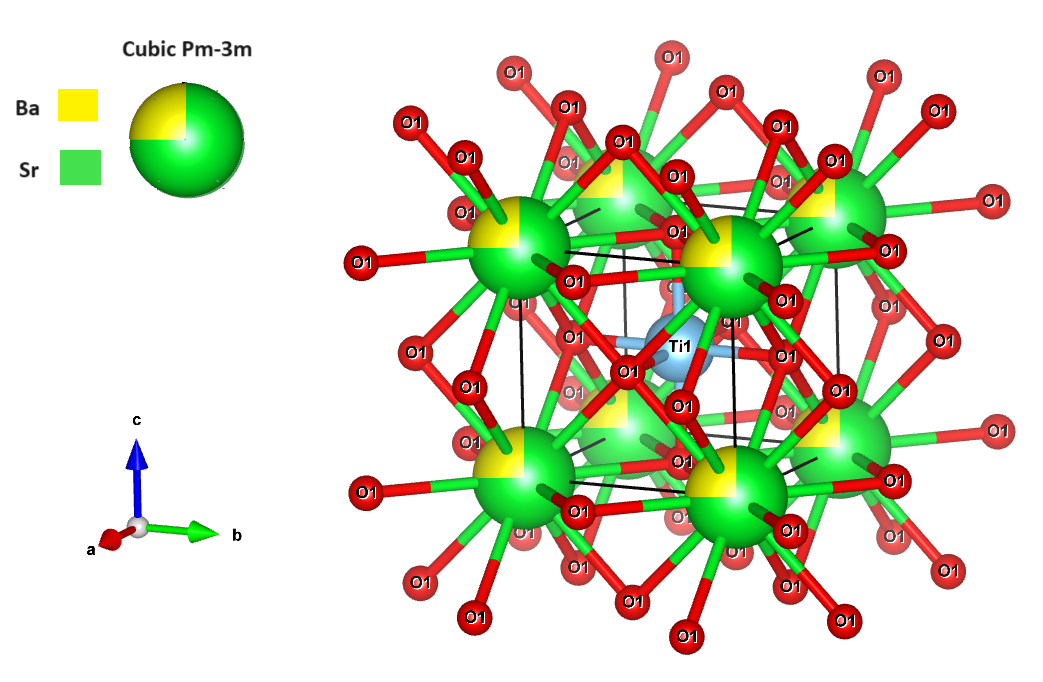 |
| **Fig. S4** Cubic crystal structure of Sr_1-x_Ba_x_TiO_3_ (0.0 < x ≤ 0.4). |
